# Biological hydrogen methanation systems – an overview of design and efficiency

**DOI:** 10.1080/21655979.2019.1684607

**Published:** 2019-11-03

**Authors:** Davis Rusmanis, Richard O’Shea, David M. Wall, Jerry D. Murphy

**Affiliations:** a MaREI Centre, Environmental Research Institute (ERI), University College Cork (UCC), Cork, Ireland; b School of Engineering, UCC, Cork, Ireland

**Keywords:** Biological methanation, biomethane, hydrogenotrophic archaea, hydrogen, methane, power to gas, gas-liquid mass transfer coefficient

## Abstract

The rise in intermittent renewable electricity production presents a global requirement for energy storage. Biological hydrogen methanation (BHM) facilitates wind and solar energy through the storage of otherwise curtailed or constrained electricity in the form of the gaseous energy vector biomethane. Biological methanation in the circular economy involves the reaction of hydrogen – produced during electrolysis – with carbon dioxide in biogas to produce methane (4H_2_ + CO_2_ = CH_4_ + 2H_2_), typically increasing the methane output of the biogas system by 70%. In this paper, several BHM systems were researched and a compilation of such systems was synthesized, facilitating comparison of key parameters such as methane evolution rate (MER) and retention time. Increased retention times were suggested to be related to less efficient systems with long travel paths for gases through reactors. A significant lack of information on gas-liquid transfer co-efficient was identified.

## Graphical Abstract

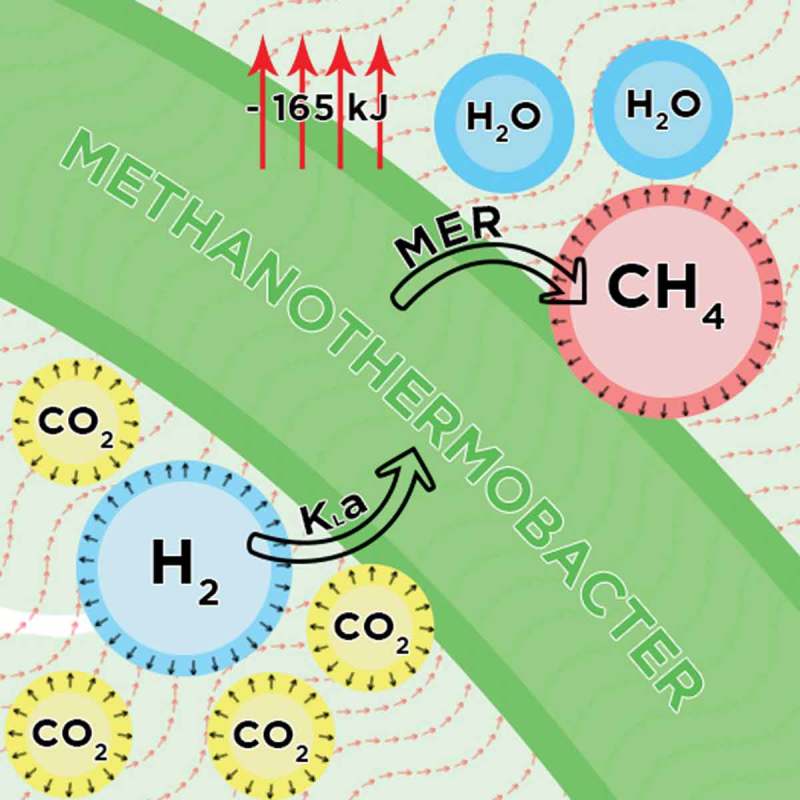

## 1. Introduction

### 1.1. Background

As part of the COP21 Paris Agreement, many countries have agreed on action to reduce greenhouse gas (GHG) emissions and meet reduction targets in order to limit global temperature rise below 2°C. It is often misconstrued that electricity can effect a silver bullet solution for decarbonizing energy with emphasis put on solar and wind energy devices. Of issue with renewable electricity is the variable intermittent nature of electricity production (from wind and solar), the temporal mismatch with variable energy demand (night and day, summer and winter), and differences in energy vectors used in each energy sector (solid, gaseous and liquid fuels for production of electricity, heat and transport). Fossil fuel systems have been optimized over hundreds of years and will be difficult to fully displace in a short period of time. Fossil fuel systems are dispatchable, easy to store, have high energy density and are cheap. Renewable energy systems are at various stages of maturity and to replace the full extent of fossil fuel systems we must overcome challenges in unpredictability, storage, and cost. Wind and solar systems are the most developed of the renewable energy systems and recently have become price comparative with fossil fuel–generated electricity, but intermittency and storage are still of issue. The energy transition will involve innovative integrated design, generating dispatchable decarbonized electricity, heat and transport fuel, including solid, liquid and gaseous vectors. Hydrogen (H_2_) generated from curtailed/constrained solar/wind energy during times of low demand/excess generation can serve as both a means of conversion to renewable gaseous fuel and as a storage mechanism through ‘Power-To-Gas’ concepts [1]. In the energy transition utilizing the natural gas infrastructure of many developed economies, biomethanation involves a circular economy integration of organic waste treatment using anaerobic digestion (AD), coupled with H_2_ production from intermittent renewable electricity. Natural gas may be substituted with biomethane and renewable gaseous fuel from non-biological sources (i.e. renewable electricity).

### 1.2. Anaerobic digestion

Many EU countries are unlikely to meet their 2020 renewable energy share (RES) targets which range from 10% RES (in Malta), to as high as 49% RES (in Sweden). In the case of Ireland, 16% of all energy must be sourced from renewables by 2020; however, as of 2017, overall RES was just 10.6%[2]. Conversion of wastes to biomethane using AD can reduce GHG production (from fugitive methane emissions) and increase RES in the form of green gas [3], reducing the current shortfall in EU targets. Significant resources of biomass including livestock manures and slurries, as well as terrestrial biomass from non-food cellulosic material, may be utilized in AD systems. AD can reduce GHG emissions by replacing open slurry storage (with significant fugitive methane emissions) with the concurrent generation of low carbon (even GHG negative) energy in the form of biogas [4], and digestate as a valuable bio-fertilizer. The uptake of AD within International Energy Agency (IEA) Bioenergy countries is significant in Germany, the UK, France, and Switzerland [5,6]. Countries such as Estonia, Ireland and Norway have a huge potential for growth in the resource of biogas. Biogas primarily consists of methane (CH_4_) (50-70%v/v) and CO_2_ (30-50%v/v) [7]. To produce biomethane, the CO_2_ is removed from the biogas. The energy content of biomethane is of the order of 35 MJ/m_STP_^3^, slightly lower than that of natural gas (39MJ/m_STP_^3^) [8]. The use of biomethane is, however, becoming more prevalent as depicted in the latest IEA Bioenergy reports [9], with rapid growth in biomethane production in the UK, France and Denmark [5]. Biogas upgrading can be considered an energy-intensive process, traditional biogas upgrading technologies have an energy demand ranging from 0.05 kWh_e_/Nm^3^ to 0.76 kWh_e_/Nm^3^ raw biogas [7,10].

### 1.3. Biological hydrogen methanation

More recently, biological hydrogen methanation (BHM) has been investigated as a means of upgrading biogas [7,11]. The process involves the Sabatier reaction, utilizing a carbon source (CO_2_) and H_2_ to generate CH_4_ (Equation 1).

Equation \ 1 $$4H_{2}+CO_{2}\to CH_{4}+2H_{2}O\Delta G^{0}=\;-165\;kJ/mol$$

The BHM process utilizes this reaction, catalyzed by specific archaea of Methanothermobacter genus, capable of converting H_2_ and CO_2_ to CH_4_ with water as a by-product. This biological method of CO_2_ conversion could potentially eliminate the traditional energy-intensive CO_2_ separation processes in AD whilst allowing for the potential doubling of the CH_4_ yield (depending on biogas composition). In turn, CH_4_ could be directly injected into the natural gas grid if grid quality specifications are met.

BHM is a means of capturing CO_2_ in biogas that would have been emitted to the atmosphere and converting to an extra quantity of renewable fuel; this is termed gaseous fuel from non-biological origin in the recast Renewable Energy Directive (RED II) [12]. As such the system can be more sustainable than biogas by itself, if the hydrogen is sourced from renewable electricity that would have been curtailed or constrained [13]. In terms of economics, the BHM system can displace conventional biogas upgrading such as water scrubbing. Thus, the net cost of the system is the cost of BHM less the saved costs of not installing traditional biogas upgrading. Also, the costs per unit of the produced gas are reduced as typically 70% extra gas is produced [11] so the investment is divided between 70% more units of energy.

**Figure 1.**
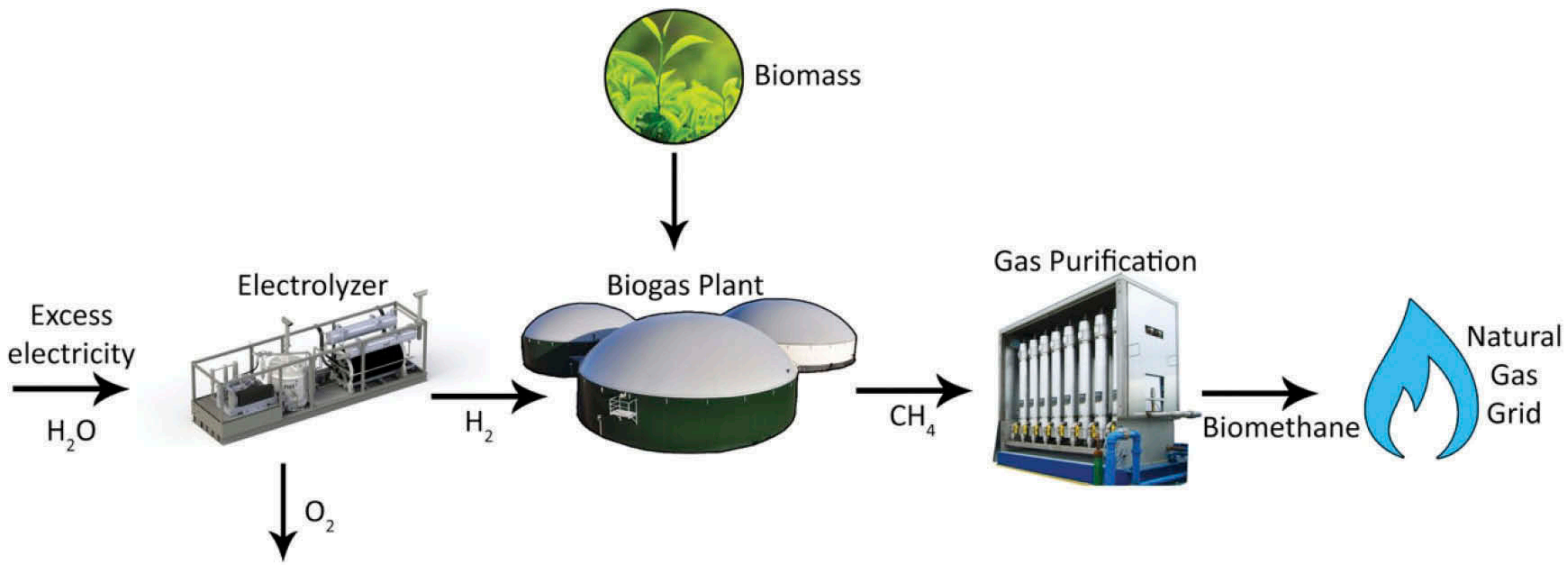
In-situ biological H_2_ methanation schematic.

The BHM process is capable of being carried out both within an anaerobic digester system known as in-situ, or in a separate, adjacent reactor known as ex-situ [7,14]. In-situ biomethanation takes place within the anaerobic digester. H_2_ gas is introduced typically through mixing or diffusion, to maximize the contact area with hydrogenotrophic methanogenic archaea, which produce CH_4_ from CO_2_ and H_2_. Standard anaerobic digestion of feedstock also occurs within the reactor, providing nutrients, contained within the digested substrates, and also CO_2_, needed by various microbes through acetogenesis, methanogenesis and methanation (Figure 1).

**Figure 2.**
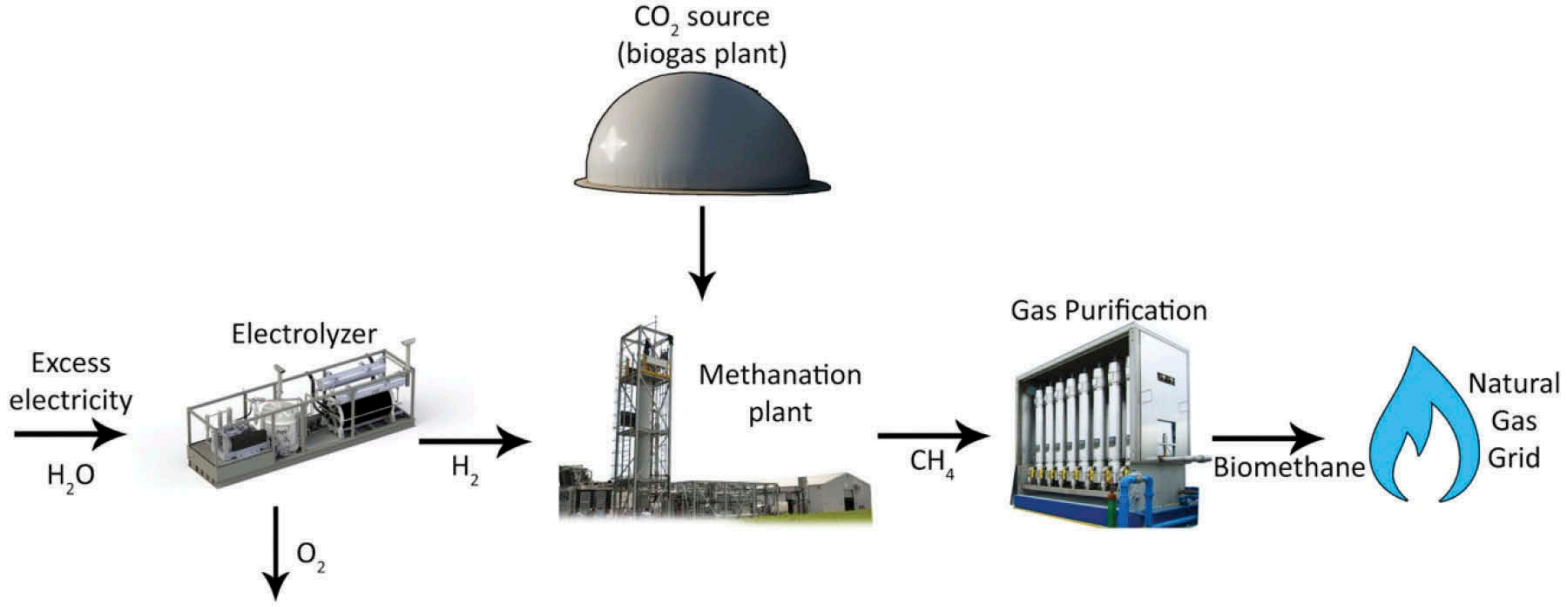
Ex-situ biological H_2_ methanation schematic.

Ex-situ methanation takes place in a separate external reactor, typically tailored to suit the hydrogenotrophic methanogens. Specific nutrient media are supplied to the microbial consortium, under a controlled environment. Gaseous reagent supply is also maintained to ensure optimal growth conditions and product concentrations. Gas purification remains to ensure grid quality gases and usually includes drying of gas to remove water vapor. (Figure 2).

BHM can be an energy-intensive process due to the processes which are required for effective H_2_ solubilization to allow H_2_ uptake by archaea, such as intense mixing from impellers, compressors and recirculation of gas and liquids. This results in higher parasitic energy demands for the upgrading process. The literature suggests that agitation as a method of H_2_ solubilization in the liquid is suitable for BHM [15–17]. However, agitation constitutes a large energy demand for these BHM systems. This is further compounded by the requirement for continuous operation, i.e. minimum power consumption of the plant can be assumed to be the power demand of the mixing component at an idle stage. At a large scale, the use of high rate agitation to promote H_2_ solubilization may be justified, but a low energy demand alternative would be far more beneficial.

The aim of this paper is to review the current literature on BHM systems with regards to system performance, H_2_ solubilization methods used in literature, and to determine the gaps in the state of the art with regards to BHM. This paper will also seek to use a short case study in order to see how biological methanation compares with traditional upgrading technologies.

## 2. Review of state of the art

The following section investigates the key operational parameters in biomethanation including the interplay between gases, microbiology, nutrient supply, reactor environmental conditions, and methane evolution rates (MER). A number of reactor configurations described in the literature are studied and compared in an effort to assess performance across systems.

### 2.1. The role of hydrogen in power to gas systems

Hydrogen gas can be generated through electrolysis which is currently the only large-scale viable method for H_2_ production that does not require fossil fuels, unlike the reformation of natural gas [18]. Hydrogen has been seen as a viable fuel for future energy systems as storage technologies become mature and more economically viable. Currently, the natural gas grid is not suitable for storing large H_2_ volumes (or convey high percentages of hydrogen in natural gas) as most pipelines would not be of the specification to prevent leakage. Small concentrations of H_2_ are allowed to be injected into the existing gas grid infrastructure in some countries [19]. However, H_2_ concentrations in gas grids are heavily regulated. In Europe, the maximum allowable H_2_ concentrations vary from 0.1% to 10% by volume and vary by country [20,21], as shown in Table 1.

**Table 1.** List of hydrogen limits allowed in the natural gas grid per country.

	Maximum H_2_ content in high pressure gas pipeline	
Country	volume/molar %	% Mass
Czech Republic^a^	0	0
Ireland^a^	0.1	0.013
UK^a^	0.1	0.013
Belgium^a^	0.1	0.013
Sweden^a^	0.5	0.063
Holland^a^	0.5	0.063
Austria^a^	4	0.524
Switzerland^a^	4	0.524
France^a^	6	0.802
Germany^a^	10	1.396
Case1^b^	2	0.256
Case 2^b^	5	0.661
Case 3^b^	10	1.396

^a^Adapted from M. Svensson[21]

^b^Cases were proposed by Altfeld and Pinchbeck [22].

Case 1 – If a CNG filling station is connected.

Case 2 – If no filling station, gas turbine or gas engines with H_2_ specification <5% connected.

Case 3 – If no filling station, gas turbine or gas engines with H_2_ specification <10% connected.

Historically, carbon steel, stainless steel, cast iron and nickel steel have been used to transport H_2_, but are not considered suitable for high-pressure H_2_ transmission [23]. Furthermore, at elevated temperatures and pressures, H_2_ attacks mild steel and other high strength steels, causing decarburization and embrittlement when in contact for extended periods of time, particularly with high purity H_2_ [20,23]. As high-quality steel is required to facilitate conveyance and storage of H_2_, a large investment is required for a H_2_ economy. Studies suggest that the optimum diameter for a transportation pipeline for 100% H_2_ is 0.88 m, while for 100% natural gas, it is 0.54 m [20]. Therefore, for a H_2_ pipeline with a wall thickness of 10 mm, 1.6 times the volume of steel would be required as compared to a natural gas pipeline.

Typically, where H_2_ is produced through electrolysis, it is compressed to high pressures, 200–300 bar for storage in gas cylinders; and up to 800 bar for use in transportation [24]. For the purpose of grid injection, costs may be lower due to gas undergoing direct compression to a grid level pressure. Compression is also an issue when using H_2_, owing to its low density (0.09 kg/m^3^). Transporting H_2_ through pipes requires a larger diameter pipe or more compression power as compared to CH_4_ to achieve the same energy throughput at the same operational pressure [20], with calculations showing that per kilometer of pipeline, the energy demand of H_2_ is significantly higher than that of CH_4_ (see Box S1 in supplementary data). Previous studies have stated that the cost of a large-scale transmission grid for H_2_ is approximately 1.5–1.8 times that of natural gas/CH_4_ [20,25].

When raw biogas from AD is upgraded to biomethane, it can be injected into the existing natural gas grid infrastructure or compressed into Liquefied Natural Gas (LNG). The existing natural gas grid provides a means of energy storage for biomethane. As such, it is viewed as a solution to the anticipated energy storage issues that may arise in countries such as Denmark who intend to fully transition away from fossil fuels and be based solely on renewables by 2050 [26, 27].

While H_2_ may be more efficient in energy density per unit mass, it is four times less dense in volume compared to CH_4_. The issues associated with the construction of H_2_ gas networks, including for material requirement and other complications relating to density may not justify the large-scale shift to a H_2_ gas grid when an existing natural gas grid may be used for biomethane. However, in some instances, localized city micro-gas grids are being built for H_2_ such as in the UK [3].

Issues relating to the transportation of H_2_ could be mitigated by using H_2_ in BHM coupled with AD. While some studies have attempted in-situ upgrading, albeit at lower efficiencies, ex-situ is more developed. Removal of CO_2_ from the raw biogas by upgrading to biomethane can almost double CH_4_ yields (if biogas is 50% CO_2_ and 100% efficiency is achieved). The extra energy obtained in the form of methane far exceeds the parasitic energy input in upgrading the gas. In essence, this is a fuel produced from biogenic CO_2_, sometimes termed gaseous fuel from non-biological origin, which is considered an advanced transport fuel within the recast Renewable Energy Directive (RED II) [12].

Hydrogen may be generated from intermittent wind and solar energy that could otherwise have been curtailed or constrained [13] and used as an energy source or used in conjunction with AD plants to upgrade raw biogas to a grid quality biomethane gas (95% CH_4_). As such, the CH_4_ produced would allow for an efficient, cost-effective energy storage of surplus/constrained electricity in the form of green gas in the natural gas grid. Integration of H_2_ has the potential for the development of circular economy systems, integration of intermittent renewable energy production and elevating the biomethane resource whilst greening the gas grid and using existing infrastructure.

### 2.2. Microbiology of BHM and AD

The Methanothermobacter genus is responsible for the biological upgrading process. To date, eight main species of this genus have been officially identified in previous literature as functional methanogens [28–32]. Table 2 lists some of the properties of Methanothermobacters. Studies on Methanobrevibacters (mesophilic archaea) are limited and do not discuss BHM applications. There could be merit in researching mesophilic methanogenic archaea for BHM applications if these archaea are capable of matching the production and doubling rates of Methanothermobacters, the lowered environmental temperature at mesophilic conditions would result in increased hydrogen solubility.

**Table 2.** Archaeal data on Methanothermobacters.

	Opt. Temp.		Chemical addition	Specific Growth rate	Doubling time	Size	
Methanothermobacter	°C	Opt. pH	g/l	/h	h	µm x µm	Reference
*Crinale*	65	6.9	0.025 NaCl	3.6	***0.193***	0.3 x 5	[28]
*Defluvii*	60	7	0.8–20 NaCl 0.025 Na2S	***0.462***	1.5	0.42 x 6	[30]
*Thermoflexum*	55	8.1	30 NaCl 0.05 Na2S	***0.198***	3.5	0.4 x 20	[29,30]
*Wolfeii*	60	7.25	0.002 Na2WO4 10 NaCl	***0.185***	3.75	0.4 x 2.5	[33]
*Tenebrarum*	70	7.3	2.5 NaCl	***0.058***	12	0.5 x 10.5	[31]
*Marburgensis*	65	7.2	0.5 NaCl	0.1	0.288	0.6 x 6	[34,65]
*Thermoautotrophicus*	67	7.4		0.231	***3***	0.6 x 7	[43]
*Thermophilus*	57	7.6	15 NaCl			0.36 x 6.5	[35]

Where values were missing from literature, highlighted results (**bold**, *italic* and underlined) are calculated using the formula for specific growth rate of microbes: *μ* = ln(N_2_/N_1_)/(*t*_2_ − *t*_1_); where *μ* is the specific growth rate, and N_1_ and N_2_ are the biomass at time 1 (*t*_1_) and time 2 (*t*_2_), respectively.

**Figure 3.**
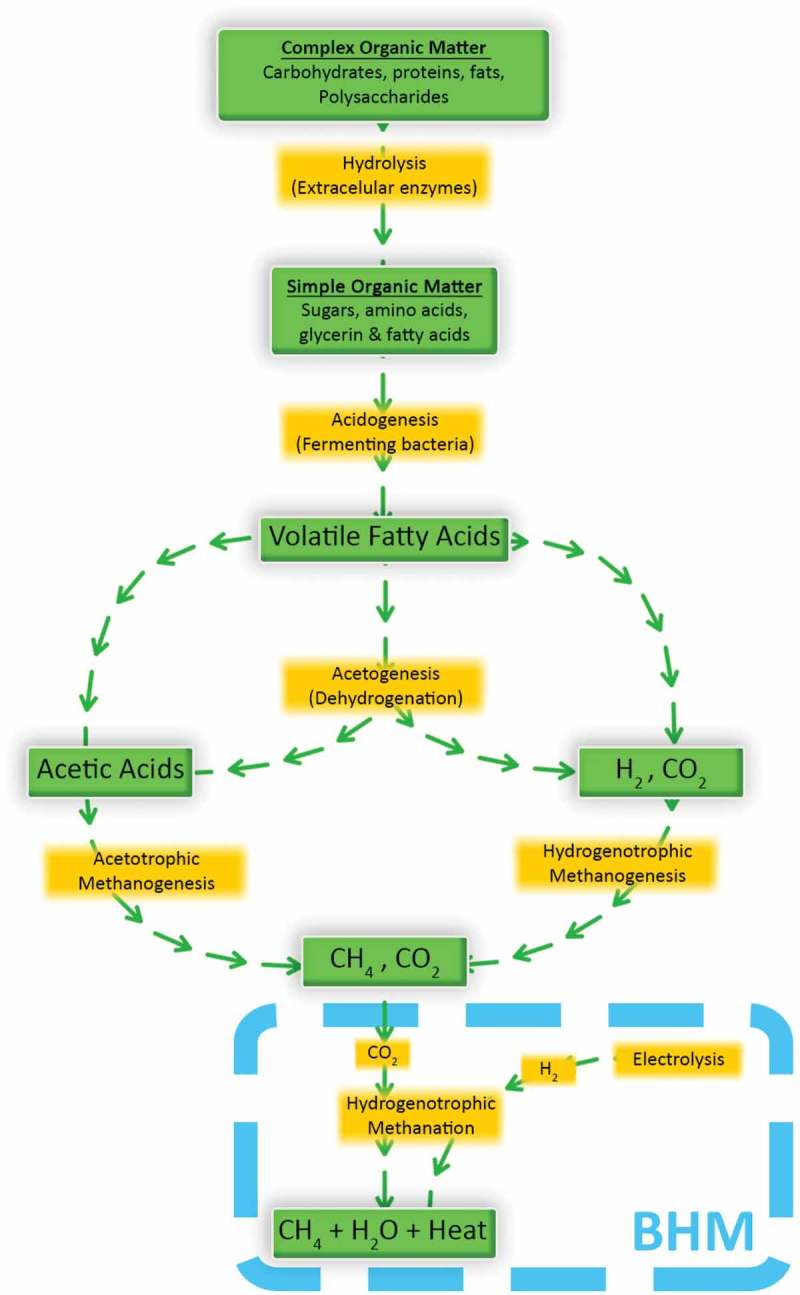
CH_4_ formation through anaerobic digestion and biological hydrogen methanation.

A summary of the biological processes which occur in the anaerobic environments of AD and BHM is illustrated in Figure 3. There are two key stages required for a BHM system to function. The first is the formation of CH_4_ and CO_2_ from the feedstock through processes known as acetogenesis and acetotrophic methanogenesis; which are responsible for the biogas production cycle in AD. The second stage involves the production of CH_4_ via H_2_ upgrading (hydrogenotrophic methanogenesis, referred to as hydrogenotrophic methanation for BHM stage). Both processes require a specific environment to be maintained, with a pH in the range of 6.2–8.5 and a temperature range of 35-40°C (mesophilic) [36], or 55-65°C (thermophilic) [37].

**Figure 4.**
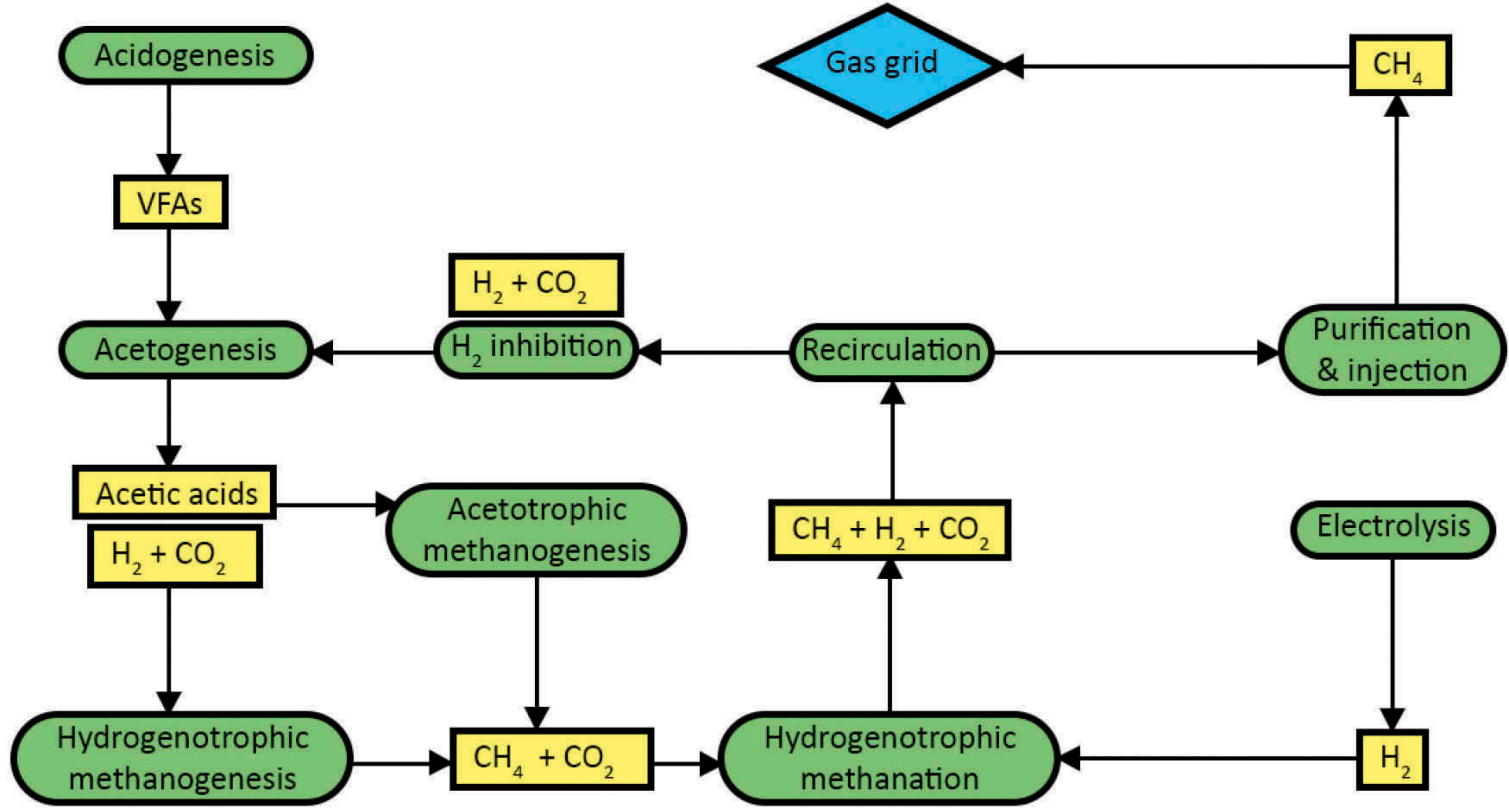
In-situ methanation failure process flow diagram.

The difference in the reactor environment for the two stages (AD and BHM) lies in the presence of H_2_ in the reactor. If an in-situ system is used and H_2_ is introduced to the AD reactor, the increase in hydrogen partial pressure can lead to inhibition of volatile fatty acid (VFAs such as propionate and butyrate) degradation and eventual potential breakdown of the system (if not micro-managed) as acetogenesis requires very low H_2_ partial pressures [38,39]. This would require an in-situ system to convert close to 100% of H_2_ injected, as lower conversion would theoretically lead to build up of unconverted H_2_ creating looping inhibition cycles, leading to reactor failure (Figure 4).

Due to differences in the required environment for acetogenesis, acetotrophic methanogenesis and hydrogenotrophic methanation, the viability of efficient in-situ systems with high Methane Evolution Rates (MER) and purity is more challenging than for ex-situ systems. While in-situ systems would reduce the need for additional construction in an AD plant, the fundamental restriction of hydrogen partial pressures within the reactor space can theoretically lead to reactor failure. However, experiments by G. Luo and I. Angelidaki [17,40] (including for hollow fiber membranes) at a small scale have been able to produce and upgrade biogas to a grid level quality at lab scale with in-situ upgrading. The experiment consisted of a 1 L reactor volume, with 4-h retention time, 1.5 barg pressure, and a second 1 L reactor volume with a 0.75 bar pressure and agitation at 150 rpm, respectively. Large-scale studies have yet to be published that depict a stable, functioning in-situ system. Thus, ex-situ systems maintaining a separate environment between acetotrophic methanogens and the biological hydrogen methanation processes are recommended by the authors; this ex-situ process allows separate conditions to be maintained, optimizing both processes and minimizing the risk of reactor failure.

Microbes that undertake methanogenesis respire anaerobically, utilizing oxidized carbon such as CO_2_ as an electron acceptor. Methanogens are found commonly in anaerobic environments which do not contain many oxygen sources, such as O_2_ or NO_3_^−^. When combining AD and biological hydrogen methanation, it is important to optimize the bioreactor to enable the desired genus to thrive and deter unwanted archaea from multiplying. In relation to sources of carbon in a BHM system, studies have shown that with a larger build up of microbial biomass, a higher fraction of CO_2_ is used by microbes due to the need for a carbon source. Burkhardt et al. [41] noted that a molar ratio for H_2_ to CO_2_ of 3.76:1 gave a higher quality gas in terms of methane content. This resulted in a reduction of available carbon sources, which could result in excess hydrogen. As such, the stoichiometric ratio (H_2_:CO_2_) is reduced to 3.76:1. While this is elucidated by Burkhardt et al. [41], further studies are required to investigate the relationship between biomass density and molar ratio of gases.

### 2.3. Nutrients in BHM

For archaea to thrive within an environment, a unique mixture of nutrients is necessary to provide key trace elements for replication. Nutrient media relating to BHM vary substantially, depending on the specific cultures and archaea species involved in H_2_ upgrading. In order to achieve high upgrading rates, an optimum nutrient feed is required to ensure no limitation from nutrient and elemental deficiencies. Table 3 shows the variation in nutrient media supplied to microbes found in the literature. This variation can be attributed to the limitation of chemical availability and complexity of nutrient media. Using the ‘BacDive’ Strain identifier for *Methanothermobacter* suggests an industrial growth culture medium of Methanobacterium growth media 141 and 119 as depicted in Table 4. The difference between literature and industry is evident in the variety of elements and concentrations recommended by the industry. As with AD [42], the addition of trace elements and nutrients also proves to be of great benefit to BHM. Such additions allow for higher culture densities to thrive and thus, boost the overall performance of the reactor significantly, by reducing microbial doubling time and thus increasing the microbial population. In ex-situ BHM, nutrient supply becomes a necessity due to the lack of solid feed addition. Excluding H_2_ and CO_2_ (or raw biogas) fed to ex-situ systems, nothing else is introduced to the reactor, and as such, there is a need for an external nutrient supply. Studies have been carried out on the methanogenic microbiology, investigating the effect of elements on the methanogens, Wolfe et al. [43, 44] specified that sulfur is necessary for biosynthetic reactions and maintenance of a low redox potential. Guneratnam et al. [32] showed that *M. wolfeii* was dominant in thermophilic ex-situ biological hydrogen methanation and suggested, as supported by Winter et al. [45], that the microbes would benefit from the addition of tungsten (W) (8 ηM). The presence of tungsten in literature is found in one paper by Schill et al. [46]. The industrial nutrient media offered by DSMZ, in Table 4 contains tungsten as a constituent of the trace element solution in 0.40 mg Na_2_WO_4_ x 2H_2_O. Observing the elemental composition of nutrient solutions used in BHM, the elements provided to the microbial population are not very different from those required for AD.

**Table 3.** Growth media of methanogenic archaea in literature.

Schill N. et al. (1996) [46]		Jean-Paul Peillex et al. (1988) [48]		Nishimura et al. (1991) [15]		M. Voelklein et al.(2019) [11]		L. Rachbauer et al. (2016) [49]	
(g/L^a^)		(g/L)		(g/L dist. H_2_O)		(g/L dist. H_2_O)		(g/L)	
Na_2_SeO_3_	1.73 x 10^−4^					Na_2_SeO_3_	5 x 10^−3^	Na_2_SeO_3_	1.26 x 10^−4^
Na2W04	2.94 x 10^−3^								
NaCl	58.4 x 10^−0^	NaCl	40 x 10^0^	NaCl	6.1 x 10^−1^	NaCl	6.1 x 10^−1^	NaCl	300.0 x 10^−3^
NH_4_Cl	6.419 x 10^0^	NH_4_CI	2.5 x 10^−1^	NH_4_Cl	1 x 10^0^	NH_4_Cl	1 x 10^0^	NH_4_Cl	300.0 x 10^−3^
Nitrilotriacetic acid (NTA)	2.29 x 10^−1^								
MgCl_2_.7H_2_O	2.21 x 10^−1^	MgCl_2_ .2H_2_O	2.75 x 10^0^					MgCl_2_ x 6H_2_O	100.0 x 10^−3^
KH_2_PO_4_	1.361 x 10^0^	KH_2_PO_4_	3 x 10^−1^	KH_2_PO_4_	3 x 10^−1^	KH_2_PO_4_	3 x 10^−1^	KH_2_PO_4_	408.0 x 10^−3^
CoCl_2_	3.25 x 10^−4^					CoCl_2_ x 6H_2_O	5 x 10^−3^	CoCl_2_ x 6H_2_O	1.0 x 10^−3^
Na_2_MoO_4_	5.15 x 10^−4^								
NiCl_2_	6.48 x 10^−4^							NiCl_2_ x 6H_2_O	3 x 10^−4^
FeSO_4_.7H_2_O	5.56 x 10^−2^								
		Fe(NH_4_)_2_(SO_4_)_2_	2 x 10^−3^	(NH_4_)_2_SO_4_	3 x 10^−1^	(NH_4_)_2_SO_4_	3 x 10^−1^		
		KCl	3.4 x 10^−1^						
		CaCl_2_ .2H_2_O	1.4 x 10^−1^					CaCl_2_ x 2H_2_O	110.0 x 10^−3^
		MgSO_4_ .7H_2_O	3.45 x 10^0^	MgSO_4_.7H_2_0	1.6 x 10^−1^	MgSO_4_.7H_2_0	1.6 x 10^−1^		
				Resazurine	1 x 10^−3^				
								Na_2_S X 9H_2_O	360.3 x 10^−3^
				CaCl_2_.2H_2_0	8 x 10^−3^				
				FeSO_4_.7H_2_O	3 x 10^−3^				
				K_2_HPO_4_	3 x 10^−1^				
				NaHCO_3_	5 x 10^0^			Na_2_HPO_4_	426.0 x 10^−3^
				Trace minerals soln.	10 x 10^0^				
				Vitamins solution	10 x 10^0^				
				Yeast extract	2 x 10^0^				
				Trypticase	2 x 10^0^				
								conc. HCl	1.0 x 10^−3^
								H_3_BO_3_	5 x 10^−5^
								ZnCl_2_	7 x 10^−5^
								CuCl_2_ x 2H_2_O	5 x 10^−5^
								MnCl_2_ x 4H_2_O	2.0 x 10^−3^
						H_24_Mo_7_N_6_O_24_ x 4H_2_O	5 x 10^−3^	(NH_4_)_6_Mo_7_O_24_ x 4H_2_O	1 x 10^−5^
								AlCl_3_ x 6H_2_O	9 x 10^−5^
								EDTA (Disodium salt)	1.0 x 10^−3^
						FeCL_3_ x 6H_2_O	5 x 10^−5^	FeCl_2_ X 4H_2_O	2.0 x 10^−3^
						Cl_2_Ni x 6H_2_O	5 x 10^−3^		

^a^ Assumed per Liter of water as not specified by author. Originally in mM/µM.

**Table 4.** DSMZ nutrient media for the Methanothermobacter genus (141. for Defluvii and thermoflexus) (119. for Crinale, Marburgensis, Tenebrarum, Thermoautotrophicus, Thermophilus, wolfeii).

141. METHANOGENIUM MEDIUM (/L dist. H_2_O)		119. METHANOBACTERIUM MEDIUM (/L dist. H_2_O)	
KCl	3.40 x 10^−1^ g	KH_2_PO_4_	5.38 x 10^−1^ g
MgCl2 x 6 H2O	4.00 x 10° g		
MgSO4 x 7 H2O	3.45 x 10° g	MgSO_4_ x 7 H_2_O	4.30 x 10^−1^ g
NH4Cl	2.50 x 10^−1^ g	NH_4_Cl	4.30 x 10^−1^ g
CaCl2 x 2 H2O	1.40 x 10^−1^ g	CaCl2 x 2 H_2_O	5.38 x 10^−2^ g
K2HPO4	1.40 x 10^−1^ g		
NaCl	1.80 x 10^1^ g	NaCl	4.30 x 10^−1^ g
Trace element solution	1.00 x 10^1^ ml	Trace element solution SL-10	1.08 x 10° ml
Fe(NH4)2(SO4)2 x 6 H2O solution	2.00 x 10° ml		
Na-acetate	1.00 x 10° g	Na-acetate	1.08 x 10° g
Yeast extract (OXOID)	2.00 x 10° g	Yeast extract (OXOID)	1.08 x 10° g
Trypticase peptone (BD BBL)	2.00 x 10° g		
Na-resazurin solution (0.1% w/v)	5.00 x 10^−1^ ml	Na-resazurin solution (0.1% w/v)	5.38 x 10^−1^ ml
NaHCO3	5.00 x 10° g	NaHCO_3_	4.30 x 10° g
Vitamin solution	1.00 x 10^1^ ml		
L-Cysteine-HCl x H2O	5.00 x 10^−1^ g	L-Cysteine-HCl x H_2_O	5.38 x 10^−1^ g
Na2S x 9 H2O	5.00 x 10^−1^ g	Na_2_S x 9 H_2_O	5.38 x 10^−1^ g
		Fatty acid mixture	2.15 x 10^1^ ml
		Na-formate	2.15 x 10° g
		Sludge fluid	5.38 x 10^1^ ml
		FeSO_4_ x 7 H_2_O solution (0.1% w/v in 0.1 N H_2_SO4)	2.15 x 10° ml
		KH_2_PO_4_	5.38 x 10^−1^ g

Archaea growth is heavily reliant on nutrient availability and the supply of gaseous feed, determined by the volumetric gas-liquid mass transfer coefficient (k_L_a) [47]. As such, gas should also be treated as an essential growth component for archaea. The higher the accessibility of nutrients and gas to microbes, the more populous the reactor and the more efficient the system could be in improving methane yields. Microbial environmental conditions are closely related to the k_L_a factor which defines the consumable H_2_ availability within the liquid [15, 48].

### 2.4. Process conditions

#### 2.4.1. Pressure

Pressure plays a key role in the increase of methanation levels [50]. The higher the molecular volume of gases such as H_2_ in a fixed volume the higher the gas solubility. The greater the number of H_2_ molecules interacting with the gas–liquid interface the greater the interphase contact. As stated by Henry [51], water under pressure of one, two or more atmospheres, absorbs a quantity of two, three or more volumes of gas that is absorbed under normal atmospheric pressure.

Increased pressure gives better gas diffusion rates due to an elevated concentration gradient across the gas and liquid phases. Hydrogenotrophic methanogenic archaea are resistant to extreme pressures of over 100 atm and exhibit improved growth and methanogenesis rates at higher pressure [52]. Many studies have managed to optimize the archaeal growth and activity within systems but are restricted by the gas to liquid transfer limitations. Most commonly, pressure is increased to mitigate this issue, allowing higher gas-liquid mass transfer rates, with minimal alteration to systems.

#### 2.4.2. Temperature

Many studies on the effect of temperature (mesophilic and thermophilic) on archaea in AD can be found in the literature [37, 53, 54]. The effect of temperature relates to two important aspects of biological methanation; the archaea growth rate and dissolution temperature dependence. In order to achieve optimum temperatures for thermophilic Methanothermobacters, temperature ranges of 55-65°C (35-40°C for mesophilic archaea) are required as outlined in Table 2. The temperature dependence of mass transfer flowrate into the liquid is also seen in Equation 2 through Henry’s law constant for H_2_. As stated by Henry [51], under isothermal and isobaric conditions, water takes up the same volume of gas. Most studies examining the temperature effects on anaerobic microbes showed that as the operational temperature is lowered, the maximum specific growth rates of archaea and substrate uptake rate decreases [55]. Variations in temperature alter the physical and chemical characteristics of reactor liquors such as viscosity. Solubility of gases also increases with lower temperatures, giving higher diffusivity levels for CH_4_, CO_2_ and H_2_ at lower temperatures. This would also mean higher CO_2_ diffusion and result in lower pH, where in the case of biomethanation, dissolution of H_2_ leads to a pH increase [56]. Diffusivity and temperature can be correlated as per equation 2;

$$\;\;\;\;\;\frac{D_{L}\mu }{T}=Constant\;\;\;(Equation\;2)$$

where D_L_ is the diffusivity of the solute at infinite dilution (cm^2^/s), µ is the viscosity of the solution, and T is the absolute temperature (K). Diffusivity, temperature and solution viscosity affect one another, also affecting the k_L_a variable. This means that k_L_a is unique for each reactor setup and is highly sensitive to the environment created for BHM. This is further discussed in Section 2.5.1.

### 2.5. Biological hydrogen methanation systems: a comparison

A comparison of the different types of systems used for BHM is presented. Various systems are compared using available key performance metrics. Calculations of these performance metrics are defined in the following sub-sections.

#### 2.5.1. Key operational parameters – k_L_a – volumetric gas-liquid mass transfer coefficient

The theory of interphase mass transfer in biological processes is well defined; however, the application of these theories has mainly focused on the oxygen transfer in the aerobic processes of wastewater treatment. In an anaerobic process, multiple gases are produced and consumed, rendering standard estimation methods challenging and/or unreliable. The volumetric gas-liquid mass transfer coefficient (k_L_a) (also referred to as the absorption coefficient [57]) is frequently mentioned throughout BHM literature, but k_L_a is rarely obtained from experimental reactors [58]. While this data is scarce in the literature, it is crucial in allowing for the determination of the efficiency of a reactor system, and for cross-system performance comparisons. k_L_a indicates the system's ability to diffuse specific gases into a liquid. Relying on multiple parameters, k_L_a is unique for a given reactor configuration and operating conditions.

The gas-liquid mass transfer rate is widely acknowledged as being the key to elevating the H_2_ upgrading rate through mixing and other forms of dissolution. Equation 3 describes the gas-liquid mass transfer rate relationship.

$$\;\;r_{t}=22.4k_{L}a\left(H_{2g}-H_{2l}\right)\;\;\;(Equation\;3)$$

Where; *r_t_ (L/L/h)* is the gas-liquid transfer rate; *22.4* is the molar volume; *k_L_a (/h)* is the gas transfer coefficient; *H_2g_* (mol/L) is the H_2_ concentration in the gas phase; *H_2l_* (mol/L) is the H_2_ concentration in the liquid phase.

Within this, k_L_a is a key coefficient, comprised of two other coefficients [59]:

k_L_: The ‘film’ coefficient, a function of the nature of the gas and of the physiochemical properties of the liquid phase [60](m/h).

a: The specific area of the interface per unit volume of liquid in the reactor (/m).

While carrying out experiments, it is key to determine k_L_a for H_2_, CO_2_ and CH_4_; H_2_ being the most important due to the necessity of the gas for biological methanation, and its inherent poor solubility. This data is scarce in the literature, but some studies have investigated similar concepts [15,48,58,61].

k_L_a can be adjusted by changing parameters such as mixing speed [17], gas recirculation [62] and H_2_ diffusion devices [40,61]. In order to accurately approximate k_L_a and the resulting mass transfer rates, the factor relies on specific experimental data being collected. The most complex data to collect is the mass transfer of H_2_ from gas to liquid phase ($\dot{m}$_H2,G -> L_). Often studies have omitted the collection of these data due to complex procedures in its determination. Work by Díaz et al. [58,61] described the acquisition and presentation of the k_L_a value. Older sources for k_L_a include works by Peillex et al. [48,63] that presented a large set of values, which were attributed to the high mixing speeds of Straight Edge and Rushton Impellers, at rates of 300-1200rpm. Research by Nishimura et al. [64] also elucidated the importance of k_L_a, being directly proportional to the gas produced, as well as the dependence of archaeal growth on the transfer of H_2_ gas to the liquid phase.

#### 2.5.2. Key operational parameters – retention time

Retention time, also known as residence time relates to the amount of time a gas spends within the system. The lower the retention time, the more compact a system achieved due to the shorter travel path of gases within the reactor system. Numerous papers have not presented actual retention time data. As per Voelklein et al. [11], equation 4 was used to estimate the values based on available literature data. The equation utilizes the averaging of volumes of gas entering and leaving the reactor system. This includes flows for CO_2_, H_2_ and CH_4_. The equation deviates noticeably with retention times extending 50 h when compared to literature-stated values. As a result, care must be taken when working with estimated retention times.

$$\;\;RT=\frac{V_{R}}{\left(F_{gas\;in}+F_{gas\;out}\right)/2}\;\;(Equation\;4)$$

Where:

RT is the retention time (unit time); V_R_ is the reactor volume (L); F_gas, out_ is the volumetric flowrate of gases leaving the reactor (L/unit time); F_gas, in_ is the volumetric flowrate of gases entering the reactor (L/unit time).

#### 2.5.3. Key operational parameters – MER

Methane evolution rate (MER) is a crucial and simple method in calculating system performance. These data relate the volume of methane produced by a unit volume of a working reactor. MER is presented in much of the literature. Some papers have also presented values such as gas throughput, which allows for the calculation of MER. Where this was the case, manipulation of equation 5 was required.

$$\;\;MER=\frac{F_{CH_{4},out}-F_{CH_{4},in}}{V_{R}}\;\;\;(Equation\;5)$$

Where: MER is the methane evolution rate (L/L_vr_/day); F_CH4, out_ is the volumetric CH_4_ flowrate leaving the reactor (L/day); F_CH4, in_ is the volumetric CH_4_ flowrate entering the reactor (L/day); V_R_ is the reactor volume (L)

### 2.6. Reactor systems: a comparison

#### 2.6.1. Continuously stirred tank reactors (CSTRs)

CSTRs are the most common type of reactors used in AD, consisting of a cylindrical reactor space. As per the name, the reactors are continuously stirred by a set of impellers or by recirculation of reactor contents which keep the reactor contents homogeneous and in motion. Agitation-oriented diffusion has proven to be amongst the most effective ways to enable hydrogenotrophic methanogenic archaea to come in contact with H_2_ [15,26,63,65]. MER ranges from experimental data of 0.86 L CH_4_/L_vr_/day, up to industry-ready 800 L CH_4_/L_vr_/day at grid injection purity (>95% CH_4_ v/v) can be found across literature and industry (Table 5). Gas retention and interface area are maximized through the reduction of bubble diameter, usually achieved through intensive mixing. CSTRs prove to be the most effective in doing this when exceeding 1200 rpm. However, at such high speeds, high energy consumption can lead to a negative energy balance.

**Table 5.** Comparison of CSTR BHM throughout literature.

	*Reactor Configuration*		*H_2_*	*CO_2_*		*CH_4 out_*	*Pressure*	*Temp.*	*Reactor Vol.*	*MER*	*Retention Time*	k_L_a		*Rate of mixing*		Gas recirculation	*Enriched Culture*		*Unit normalized to*	
*type*	*(in/ex-situ)*	*pH*	*(L/L_vr_/min)*	*(L/L_vr_/min)*	*H_2_/CO_2_ Ratio*	*(%)*	*(Barg)*	*(°C)*	*(L)*	*(L CH_4_/L_vr_/day)*	h	/h	Method of k_L_a Increase	*(L/min)*	*(rpm)*	(L/min)	*(y/n)*	*Archaeal strain*	*(STP/NTP/NA)*	*Author*
CSTR	Ex-situ	6.8	0.30	0.08	4 : 1	2	0	60	16	33.3	0.051		Agitator	-	1000	0	Yes	M. Thermoautotrophicus	STP	[16]
CSTR	Ex-situ	6.85	0.23	0.06	4 : 1	65	0.75	65	10	75.3	0.090		Agitator	-	1500	0	Yes	M. Marburgensis	STP	[65]
CSTR	Ex-situ	6.85	0.34	0.09	4 : 1	58	0.75	65	10	107.5	0.059		Agitator	-	1500	0	Yes	M. Marburgensis	STP	[65]
CSTR	Ex-situ	6.85	0.58	0.14	4 : 1	22	0.75	65	10	123.6	0.030		Agitator	-	1500	0	Yes	M. Marburgensis	STP	[65]
CSTR	Ex-situ	6.85	1.40	0.35	4 : 1	30	5	65	10	344.1	0.013		Agitator	-	1500	0	Yes	M. Marburgensis	STP	[65]
CSTR	Ex-situ	6.85	0.40	0.10	4 : 1	80	5	65	10	137.1	0.054		Agitator	-	1500	0	Yes	M. Marburgensis	STP	[65]
CSTR	Ex-situ	-	0.89	0.22	4 : 1	99	8.5	62.5	3750	800	0.050		Agitator	-	N/A	0	Yes	Patented Strain		[26,85,86]
CSTR	In-situ	7.8	0.0013	0.0003	4 : 1	94	1.5	55	1	0.5	***8***	7	Agitator	-	500	0	Yes	-	STP	[69]
CSTR	In-situ	7.8	0.0025	0.0006	4 : 1	95	1.5	55	1	0.9	***4***	11	Agitator	-	500	0	Yes	-	STP	[69]
CSTR	In-situ	7.8	0.01	0.0013	4 : 1	90	1.5	55	1	1.6	***2***	20	Agitator	-	500	0	Yes	-	STP	[69]
CSTR	In-situ	7.8	0.01	0.0013	4 : 1	94	1.5	55	1	1.6	***2***	21	Agitator	-	800	0	Yes	-	STP	[69]
CSTR	In-situ	7.8	0.01	0.0025	4 : 1	91	1.5	55	1	3.2	***1***	40	Agitator	-	800	0	Yes	-	STP	[69]
CSTR	Ex-situ	7.4	1.60	0.40	4 : 1	18	0	65	1	300.0	0.011		Agitator	-	1300	0	No	M. Thermoautotrophicus	N/A	[15]
CSTR	Ex-situ	7.4	1.60	0.40	4 : 1	9	0	65	1	194.4	0.010		Agitator	-	1300	0	No	M. Thermoautotrophicus	N/A	[15]
CSTR	Ex-situ	6.85	0.03	0.01	4 : 1	85	0	60	3.5	9.9	0.760		Agitator	-	700	0	Yes	M. Thermoautotrophicus	N/A	[70]
CSTR	Ex-situ	6.85	0.06	0.014	4 : 1	64	0	60	3.5	18.5	0.364		Agitator	-	700	0	Yes	M. Thermoautotrophicus	N/A	[70]
CSTR	Ex-situ	6.85	0.11	0.03	4 : 1	49	0	60	3.5	34.0	0.174		Agitator	-	700	0	Yes	M. Thermoautotrophicus	N/A	[70]
CSTR	Ex-situ	6.85	0.23	0.06	4 : 1	22	0	60	3.5	47.9	0.076		Agitator	-	700	0	Yes	M. Thermoautotrophicus	N/A	[70]
CSTR	Ex-situ	6.85	0.46	0.11	4 : 1	6	0	60	3.5	40.4	0.032		Agitator	-	700	0	Yes	M. Thermoautotrophicus	N/A	[70]
CSTR	Ex-situ	6.85	0.17	0.04	4 : 1	42	0.2	60	3	46.9	0.116		Agitator	-	700	0	Yes	M. Thermoautotrophicus	N/A	[70]
CSTR	Ex-situ	7.35	0.33	0.08	4 : 1	17	0.2	60	3	61.2	0.050		Agitator	-	700	0	Yes	M. Thermoautotrophicus	N/A	[70]
CSTR	Ex-situ	7.35	0.67	0.17	4 : 1	7	0.2	60	3	65.7	0.022		Agitator	-	700	0	Yes	M. Thermoautotrophicus	N/A	[70]
CSTR	Ex-situ	7.35	0.13	0.03	4 : 1	45	0.2	60	3	38.6	0.147		Agitator	-	700	0	Yes	M. Thermoautotrophicus	N/A	[70]
CSTR	Ex-situ	6.85	0.27	0.07	4 : 1	18	0.2	60	3	50.4	0.063		Agitator	-	700	0	Yes	M. Thermoautotrophicus	N/A	[70]
CSTR	Ex-situ	6.85	0.53	0.13	4 : 1	5	0.2	60	3	38.4	0.027		Agitator	-	700	0	Yes	M. Thermoautotrophicus	N/A	[70]
CSTR	Ex-situ	6.85	0.07	0.02	4 : 1	74	0.2	60	3	16.1	0.131		Agitator	-	700	0	Yes	M. Thermoautotrophicus	N/A	[70]
CSTR	Ex-situ	6.85	0.13	0.03	4 : 1	63	0.2	60	3	32.7	0.089		Agitator	-	700	0	Yes	M. Thermoautotrophicus	N/A	[70]
CSTR	Ex-situ	6.85	0.53	0.13	4 : 1	13	0.2	60	3	26.4	0.024		Agitator	-	700	0	Yes	M. Thermoautotrophicus	N/A	[70]
CSTR	Ex-situ	7.4	4	1	4 : 1	4	1	65	1	233.3	0.004		Agitator	-	1300	0	Yes	KN-15	STP	[64]
CSTR	Ex-situ	7.4	4	1	4 : 1	9	2	65	1	460.2	0.004		Agitator	-	1300	0	Yes	KN-15	STP	[64]
CSTR	Ex-situ	7.4	4	1	4 : 1	15	3	65	1	688.1	0.004		Agitator	-	1300	0	Yes	KN-15	STP	[64]
CSTR	Ex-situ	6.8	0.52	0.13	4 : 1	42	0	65	1.5	146.7	0.037	1200	Rushton Impeller	-	320	0	Yes	M. Thermoautotrophicus	N/A	[48]
CSTR	Ex-situ	6.8	0.64	0.16	4 : 1	52	0	65	1.5	194.5	0.031	1450	Straight blade	-	320	0	Yes	M. Thermoautotrophicus	N/A	[48]
CSTR	Ex-situ	6.8	1.92	0.48	4 : 1	44	0	65	1.5	551.0	0.010	3550	Straight blade	-	660	0	Yes	M. Thermoautotrophicus	N/A	[48]
CSTR	Ex-situ	6.8	0.54	0.13	4 : 1	24	0	65	1.5	118.1	0.033	1100	Rushton Impeller	-	660	0	Yes	M. Thermoautotrophicus	N/A	[48]
CSTR	Ex-situ	6.8	1.36	0.34	4 : 1	32	0	65	1.5	334.0	0.013	3250	Rushton Impeller	-	1015	0	Yes	M. Thermoautotrophicus	N/A	[48]
CSTR	Ex-situ	6.8	1.68	0.42	4 : 1	40	0	65	1.5	465.3	0.011	3750	Straight blade	-	1015	0	Yes	M. Thermoautotrophicus	N/A	[48]
CSTR	Ex-situ	6.8	0.83	0.21	4 : 1	35	0	65	1.5	219.2	0.023		Straight blade	-	400	0	Yes	M. Thermoautotrophicus	N/A	[48]
CSTR	Ex-situ	6.8	0.83	0.21	4 : 1	76	0	65	1.5	229.0	0.023		Rushton Impeller	-	300	0	Yes	M. Thermoautotrophicus	N/A	[48]
CSTR	Ex-situ	6.8	0.83	0.21	4 : 1	43	0	65	1.5	229.2	0.023		Straight blade	-	490	0	Yes	M. Thermoautotrophicus	N/A	[48]
CSTR	Ex-situ	6.8	0.83	0.21	4 : 1	83	0	65	1.5	249.9	0.024		Rushton Impeller	-	660	0	Yes	M. Thermoautotrophicus	N/A	[48]
CSTR	Ex-situ	6.8	0.83	0.21	4 : 1	52	0	65	1.5	250.4	0.024		Straight blade	-	660	0	Yes	M. Thermoautotrophicus	N/A	[48]
CSTR	Ex-situ	6.8	0.83	0.21	4 : 1	90	0	65	1.5	270.1	0.025		Rushton Impeller	-	900	0	Yes	M. Thermoautotrophicus	N/A	[48]
CSTR	Ex-situ	6.8	0.83	0.21	4 : 1	61	0	65	1.5	271.2	0.025		Straight blade	-	850	0	Yes	M. Thermoautotrophicus	N/A	[48]
CSTR	Ex-situ	6.8	0.83	0.21	4 : 1	97	0	65	1.5	289.8	0.026		Rushton Impeller	-	1200	0	Yes	M. Thermoautotrophicus	N/A	[48]

- Retention time values presented in papers are noted (**Bold**, underlined and *italic*), but where missing, retention time according to Voelklein et al. [11] calculated to allow comparison.

Reduction of bubble diameter is achieved when bubbles break apart due to surface tension forces of the bubble interface being overcome by a higher power density. CSTR systems depend on high impeller rotational speeds in order to increase gas diffusivity. While this is an effective method of increasing the volumetric gas-liquid mass transfer coefficient (k_L_a), the energy demand of impeller agitation, such as a Rushton impellers, results in significant energy consumption by the system [61], often rendering the process inefficient. Other issues that arise with the Rushton Impellers include them being only effective in the vertical column they occupy. Scale-up would require reactor systems to have a large height to width ratio. This was elucidated by Savvas et al. [66], where a 2-L reactor with a Rushton impeller at 1200rpm had a parasitic energy demand of 7%. Scaling up of the 2-L reactor to 5 L showed a 90% parasitic energy demand of the total energy generated. Due to the turbulent nature of stirring gas-liquid contactors, power usage by the impellers is obtained using equation 6.

$$\;\;\;P=P_{0}\rho N^{3}D^{5}\;\;\;\;\;(Equation\;6)$$

Where: P_0_ is the power number, depending on structural characteristic of mixing system (geometry); ρ is the fluid density; N is the angular velocity of impeller; D is the diameter of the impeller.

Increasing the reactor radius results in a significant increase in mixing power consumption (parasitic energy demand) as the impeller power consumption increases proportionally to the diameter raised to the power of 5, as evident in Equation 6. This shows the importance of geometry of impellers also playing a crucial role in reactor performance.

The effect of high shear force exertion due to the impeller has also been raised. Such shear forces form when liquids move at different velocities. Due to the nature of impellers, velocity by the impeller tip will be larger than that near the center. While no clear inhibitions can be deduced as of yet, cell damage may be a possibility due to high shear forces. Cases have shown syntrophic interaction across groups to be limited by shear forces in CSTRs [67]. Some studies have exhibited upper limits for mixing speeds, where biogas production declines [68]. A comparison of the performance indicators of CSTR reactors used in BHM systems is shown in Table 5. Only a few reactor configurations achieve a grid injection level gas quality. These systems are either achieving this by sacrificing higher MER for high gas purity, such as Luo and Angelidaki [69] while also utilizing extended retention times for purity of gas. Other reactors such as those by Peillex et al. [48] or ‘Electrochaea GmbH’ [26] achieve high purity by using high mixing rates, elevated pressures and pure strain archaea cultures.

#### 2.6.2. Diffusion-based reactors

Diffusion as a phenomenon has been studied extensively, with research moving to micro (ø40-600µm) and nano-bubble (ø500 nm) production at viable levels [60,71,72]. These studies outline some of the common issues that must be overcome in order to achieve this scale of gas diffusion. When looking closer at the bubbles on an individual level, the interface of a bubble is governed by the Young–Laplace Law. Research found on diffusion-based reactors is limited, especially with respect to the determination of optimal diffusion rates. Few papers have studied this method of H_2_ solubilization. To the authors' knowledge, no research has focused on the bubble dynamics of H_2_ or CO_2_ in an AD or BHM setting. Voelklein et al. [11] and Bassani et al. [59] are the only papers found to study plate diffuser equipment to any appreciable degree, but micro bubble diffusion per se was not achieved. A MER range of 1.05–9.1 L CH_4_/L_vr_/day was achieved, but the CH_4_ purity was below 95%. Voelklein et al. [11] were able to achieve a grid injection purity in terms of CH_4_ content but this required a 24hr batch duration. Luo and Angelidaki [73] achieved MER of approximately 1.4L/L_vr_/day and made an estimation of the k_L_a value, presenting a very low range of 6-16/h. This is not comparable to other literature, such as that presented by Peillex et al. [48] which reached up to 3750/h. A comparison of the performance indicators of diffusion-based reactors used in BHM systems is shown in Table 6.

**Table 6.** Comparison of diffusion BHM throughout literature.

	*Reactor Configuration*		*H_2_*	*CO_2_*		*CH_4 out_*	*Pressure*	*Temp.*	*Reactor Vol.*	*MER*	*Retention Time*	k_L_a		*Rate of mixing*		Gas recirculation	*Enriched Culture*		*Unit normalized to*	
*type*	*(in/ex-situ)*	*pH*	*(L/L_vr_/min)*	*(L/L_vr_/min)*	*H_2_/CO_2_ Ratio*	*(%)*	*(Barg)*	*(°C)*	*(L)*	*(L CH_4_/L_vr_/day)*	h	/h	Method of k_L_a Increase	*(L/min)*	*(rpm)*	(L/min)	*(y/n)*	*Archaeal strain*	*(STP/NTP/NA)*	*Author*
CSTR	In-situ	7.93	0.0037	0.0009	4 : 1	6	0.2	55	9.5	0.3	***24***		Fish stone	4	-	4	No	-	STP	[11]
CSTR	Ex-situ	8.2	0.0038	0.0011	3.4 : 1	85	0.2	55	9.5	0.9	***1.63***		Ceramic Diffuser	4	-	4	No	-	STP	[11]
CSTR	Ex-situ	8.5	0.0051	0.0013	4 : 1	92	0.2	55	9.5	1.7	***24***		Ceramic Diffuser	4	-	4	No	-	STP	[11]
CSTR	Ex-situ	8.1	0.0095	0.0024	3.9 : 1	61	0.2	55	9.5	1.7	***1.14***		Ceramic Diffuser	4	-	4	No	-	STP	[11]
CSTR	In-situ	7.97	0.0035	0.0009	4 : 1	32	0.2	55	9.5	1.8	***24***		Fish stone	4	-	4	No	-	STP	[11]
CSTR	In-situ	8.37	0.0037	0.0009	4 : 1	60	0.2	55	9.5	2.5	***24***		Fish stone	4	-	4	No	-	STP	[11]
CSTR	Ex-situ	8.1	0.0102	0.0026	4 : 1	49	0.2	55	9.5	2.9	***1.99***		Ceramic Diffuser	4	-	4	No	-	STP	[11]
CSTR	In-situ	8.5	0.0107	0.0027	4 : 1	96	0.2	55	9.5	3.7	***24***		Ceramic Diffuser	4	-	4	No	-	STP	[11]
CSTR	Ex-situ	8.2	0.0351	0.0088	4 : 1	23	0.2	55	9.5	5.1	***0.41***		Ceramic Diffuser	4	-	4	No	-	STP	[11]
CSTR	Ex-situ	7.1	0.0327	0.0082	4 : 1	32	0.2	55	9.5	8.2	***0.57***		Ceramic Diffuser	4	-	4	No	-	STP	[11]
CSTR	Ex-situ	7.4	0.0509	0.0127	4 : 1	15	0.2	55	9.5	9.1	***0.33***		Ceramic Diffuser	4	-	4	No	-	STP	[11]
CSTR	In-situ	7.74	0.0022	0.0006	4 : 1	53	0	55	1	1.4	14.42	6.62	Column Diffuser	-	150	0	No	-	STP	[73]
CSTR	In-situ	7.84	0.0012	0.0003	4 : 1	68	0	55	1	1.2	92.37	11.78	Column Diffuser	-	300	0	No	-	STP	[73]
CSTR	In-situ	7.89	0.0021	0.0006	4 : 1	75	0	55	1	1.2	12.11	16.05	Ceramic Diffuser	-	150	0	No	-	STP	[73]

- Retention time values presented in papers are noted (**Bold**, underlined and *italic*), but where missing, retention time according to Voelklein et al. [11] calculated to allow comparison.

Currently, it is difficult to portray diffusion as a competitive alternative as these methods are still in their infancy. In theory, the use of microbubble diffusers could be effective in providing a large interference area between gas and archaea, without the need for packing or high energy demanding mixing, but further experiments need to be undertaken to verify this hypothesis.

Diffusion relies on the quality and type of diffuser used. This determines the dissolution of H_2_ into the liquid. When nano-scale bubbles are formed, they exhibit unique properties when compared to standard bubbles. Nano-bubbles are capable of staying suspended in a liquid, remaining in agitation through Brownian motion for extended periods of time (days), disappearing due to diffusion of the gaseous content [74]. There are some complications with this however as the generation of these nano-bubble types may require considerable energy demand if systems are not optimized properly. Several companies have developed devices which are capable of creating microbubbles, with the gas flow rates being adequate for research purposes. The current proposed concepts in the industry are:

1.Swirl-type liquid flow method [75]. This method uses a specially designed chamber, utilizing liquids rotating in a whirlpool manner to a narrower outlet. Control of this method is regulated by the supply of gaseous flowrates to the chamber. Patents by H. Ohnari [76] state that the invention is capable of generating micro-bubbles with diameters no larger than 20 µm on an industrial scale.

2.High-pressure dissolution method [77] uses elaborate system setup, increasing pressures and temperatures, followed by pressure decreases to stimulate gaseous diffusion into a liquid solute.

3.Venturi methods are now commercially available devices, utilizing the shearing of bubbles through the circulation of water in a Venturi meter-like flow device. These devices have been studied in depth and bubble diameters are varied by restraining the supply rate of the gas, resulting in a trade-off of gaseous flowrate to bubble diameter.

4.Supersonic vibration method [78] has been a long established method for increased rates of gaseous dissolution in liquids. Using designs based on a Galton whistle for air vibrations and ‘Langevin sandwich’ – comprising of currents passed through special quartz slabs, allowing for sending out ultrasonic vibrations into liquid media.

5.Ultrafine pore diffusion methods [60,72,75] utilize specially designed membranes or diffusion devices, utilizing nanopore materials (no greater than 1 µm diameter) which are used to pass gas through, generating nanobubbles. These devices can range from ceramic diffusers and hollow fiber membranes to more unique and carbon-based diffusion devices.

**Figure 5.**
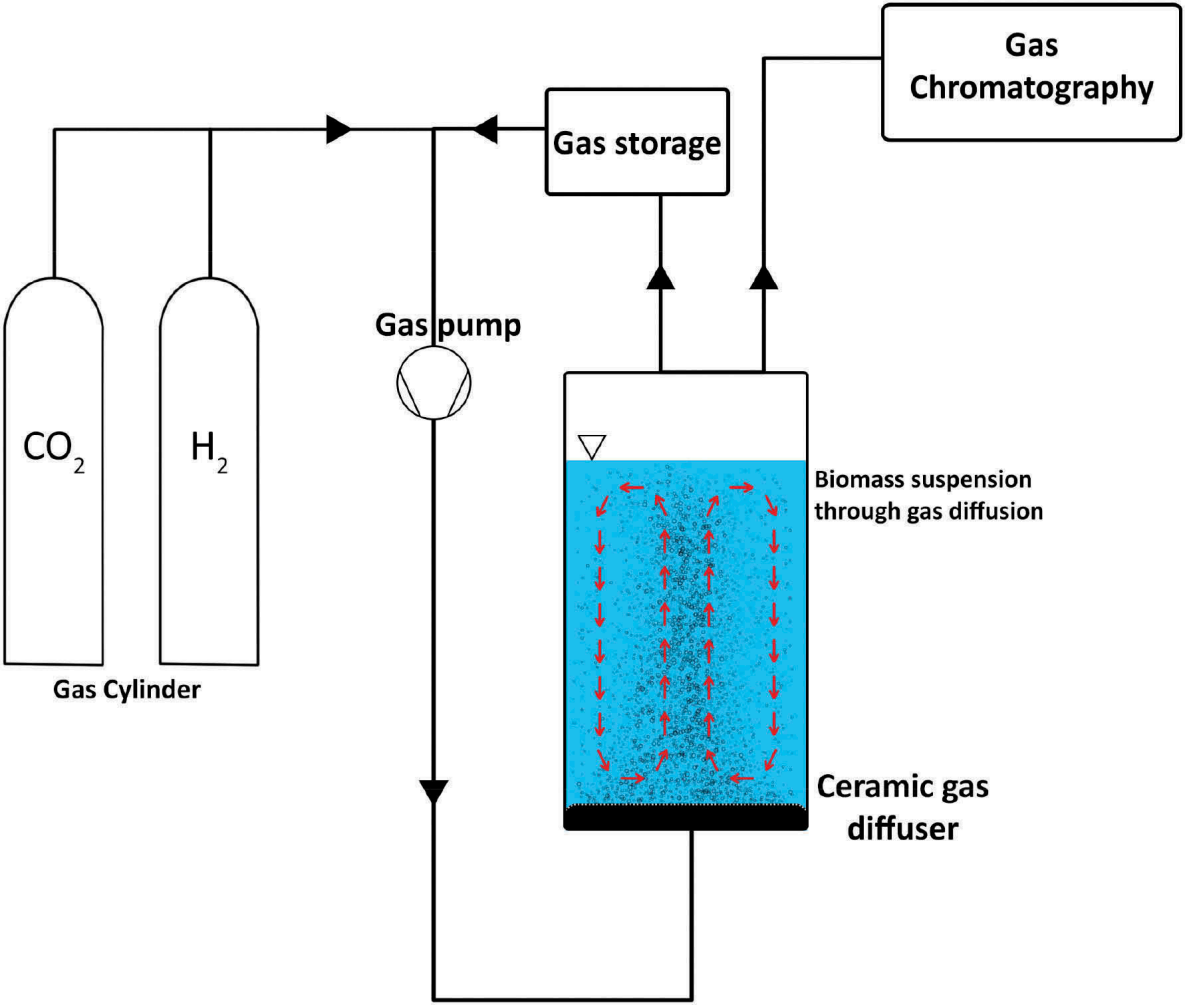
Modified CSTR with a ceramic diffuser base.

The different methods of diffusion are used for a variety of services such as aquaponics, sterilization, cleaning, environmental purification, wastewater treatment, and paper manufacture. However, the idea of applying nano-bubble technology for H_2_ dissolution, and furthermore BHM, to the best of the authors’ knowledge, has not been studied. This means a significant gap in the state of the art for BHM is open to investigation as is the viability of nano-bubble diffusion in a BHM environment. Since the system is in its infancy, the reactor topology can be varied to suit the nature of the diffusion method used. This can be carried out by a bubble column type reactor, or an entirely new concept unique to the method. In literature, a basic, modified CSTR was used, where the stirring mechanism was replaced by a ceramic gas diffuser place covering the base of the reactor. Figure 5 was adapted from Voelklein et al. [11].

Other reactor system layouts using diffusion in BHM applications are possible and should be assessed in further work.

#### 2.6.3. Fixed film bioreactors

**Figure 6.**
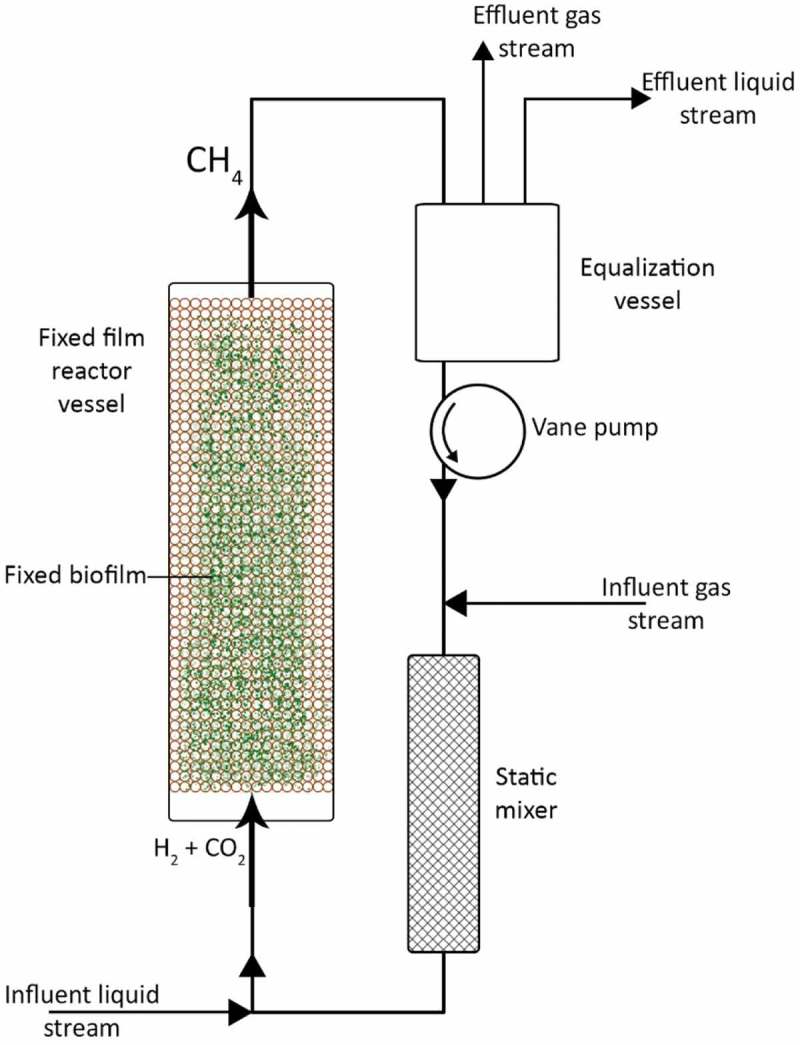
Fixed film/Anaerobic Filter and Soil packed reactor layout.

Fixed film bioreactors are set-up where the microbial populations are adhered to a packing material within the reactor such that the maximum surface area is achieved. The purpose of this is to force the maximum interference of liquid and gas. Gas is diffused from the reactor bottom and passed through the packing fixed film layer (Figure 6). Liquids are recirculated and injected in the bottom of the reactor and are allowed to percolate upwards through the packing film with the aid of the influent gas stream, providing nutrients to the archaea. In some cases, both liquid and gas are recirculated, requiring a liquid/gas separation a point before pumping [66]. A comparison of fixed film BHM reactors in varied stages of development is shown in Table 7.

**Table 7.** Comparison of Fixed Film reactors in BHM throughout literature.

	*Reactor Configuration*		*H_2_*	*CO_2_*		*CH_4 out_*	*Pressure*	*Temp.*	*Reactor Vol.*	*MER*	*Retention Time*	k_L_a		*Rate of mixing*		Gas recirculation	*Enriched Culture*		*Unit normalized to*	
*type*	*(in/ex-situ)*	*pH*	*(L/L_vr_/min)*	*(L/L_vr_/min)*	*H_2_/CO_2_ Ratio*	*(%)*	*(Barg)*	*(°C)*	*(L)*	*(L CH_4_/L_vr_/day)*	h	/h	Method of k_L_a Increase	*(L/min)*	*(rpm)*	(L/min)	*(y/n)*	*Archaeal strain*	*(STP/NTP/NA)*	*Author*
Biofilm	Ex-situ	-	0.0833	0.0208	4 : 1	90	0	37	0.75	30	0.27		Fixed Film Biomethanation	-	-	0	Yes	-	STP	[66]
Biofilm	Ex-situ	-	0.1278	0.0319	4 : 1	50	0	37	0.75	40	0.16		Fixed Film Biomethanation	-	-	0	Yes	-	STP	[66]
Biofilm	Ex-situ	-	0.0794	0.0199	4 : 1	75	0	37	0.75	27	0.27		Fixed Film Biomethanation	-	-	0	Yes	-	STP	[66]
Biofilm	Ex-situ	-	0.0578	0.0144	4 : 1	97	0	37	0.75	20	0.37		Fixed Film Biomethanation	-	-	0	Yes	-	STP	[66]
UASB	In-situ	8.18	0.0057	0.0014	4 : 1	40	0	55	1.4	1.5	2.82		Rashig Rings	-	-	0	No	-	STP	[59]
UASB	In-situ	8.38	0.0058	0.0015	4 : 1	45	0	55	1.4	1.5	2.76		Rashig Rings	-	-	0	No	-	STP	[59]
UASB	In-situ	8.24	0.0057	0.0014	4 : 1	52	0	55	1.4	1.5	2.83		Ceramic Sponge	-	-	0	No	-	STP	[59]
UASB	In-situ	7.83	0.0054	0.0014	4 : 1	66	0	55	1.4	2.1	3.60		Ceramic Sponge	-	-	0.006	No	-	STP	[59]
UASB	In-situ	7.93	0.0055	0.0014	4 : 1	66	0	55	1.4	1.8	3.18		Ceramic Sponge	-	-	0.006	No	-	STP	[59]
UASB	In-situ	7.9	0.0055	0.0014	4 : 1	68	0	55	1.4	1.8	3.38		Serial Chambers	-	-	0.006	No	-	STP	[59]
UASB	In-situ	7.92	0.0055	0.0014	4 : 1	81	0	55	1.4	1.8	3.29		Single chamber column	-	-	0.004	No	-	STP	[59]
TBR	Ex-situ	7.3	0.0034	0.0008	4 : 1	98-100	0	37	88	1.2	***4***		Trickle bed/Packing	-	-	0	Yes	-	STP	[41]
TBR	Ex-situ	7.3	0.0042	0.0010	4 : 1	98	0	37	88	1.5	***4***		Trickle bed/Packing	-	-	0	Yes	-	STP	[41]
TBR	Ex-situ	7.5	0.0038	0.0009	4.4 : 1	59	0	37	7.54	1.1	***2.9***		Packing & liquid spraying	-	-	0	Yes	-	STP	[49]
TBR	Ex-situ	7.5	0.0040	0.0006	6.8 : 1	98	0	37	7.54	0.9	***3***		Packing & liquid spraying	-	-	0	Yes	-	STP	[49]
TBR	Ex-situ	7.5	0.0038	0.0005	7.1 : 1	97	0	37	7.54	0.8	***3.2***		Packing & liquid spraying	-	-	0	Yes	-	STP	[49]
AF	Ex-situ	7.4	0.0215	0.0052	4 : 1	15	0	55	0.5	6.7	0.96		Cristobalite PQ10 + Recirculation	0.3	-	0.3	Yes	-	STP	[80]
AF	Ex-situ	6.6	0.0347	0.0087	4 : 1	16	0	65	0.083	127.2	-		Fixed bed reactor	-	-	0	Yes	M. Thermoautotrophicus	N/A	[81]
AF	Ex-situ	6.6	0.0427	0.0107	4 : 1	58	0	65	0.136	122.7	-		Fixed bed reactor	-	-	0	Yes	M. Thermoautotrophicus	N/A	[81]
AF	Ex-situ	6.8	0.0138	0.0035	4 : 1	46	0	55	4	4.0	***144***		Solid packing	-	-	0	No	-	N/A	[79]
AF	Ex-situ	6.9	0.0076	0.0019	4 : 1	26	0	55	4	1.7	***144***		Solid packing	-	-	0	No	-	N/A	[79]
AF	Ex-situ	6.9	0.0049	0.0012	4 : 1	87	0	55	4	1.7	***144***		Solid packing	-	-	0	No	-	N/A	[79]

- Retention time values presented in papers are noted (**Bold**, underlined and *italic*), but where missing, retention time according to Voelklein et al. [11] calculated to allow comparison.

Work by Bassani et al. [59] compares the performance of two reactors, one with in-situ BHM and another acting as a control AD reactor. The reactor with in-situ methanation had a lower CH_4_ concentration in the outlet gas, owing to the presence of unconverted H_2_. This is a common issue across the literature as the dissolution of H_2_ is the bottleneck for high H_2_ conversion rates. Bassani et al. [59] highlight a reactor with rashig rings with a CH_4_ outlet percentage of 40.4%, where the control AD presents 60.6%. However, the MER rates, of the in-situ and control AD were 1.528 L/L_vr_/day and 1.350 L/L_vr_/day, respectively. This highlights the problem encountered by many existing systems, use of BHM results in the reduction in CH_4_ purity leaving the reactor, but the MER is larger than the standard AD process.

This trade-off can be observed in Burkhardt et al. [41], where an 88 L reactor system achieved 98-100% CH_4_ purity, at a lower MER to that of Bassani et al. [59], 1.2–1.5 L/L_vr_/day. It is important to observe that the residence time reported by Burkhardt is significantly longer. This also applies for work by Alitalo et al. [79] who depict the reactor's inability to convert the H_2_ gas even at large batch retention times of 144 h. This could be due to the lack of an enriched nutrient supply, or lack of pure strain culture use, making high rate methanation difficult to achieve.

#### 2.6.4. Minimal liquid bioreactor

**Figure 7.**
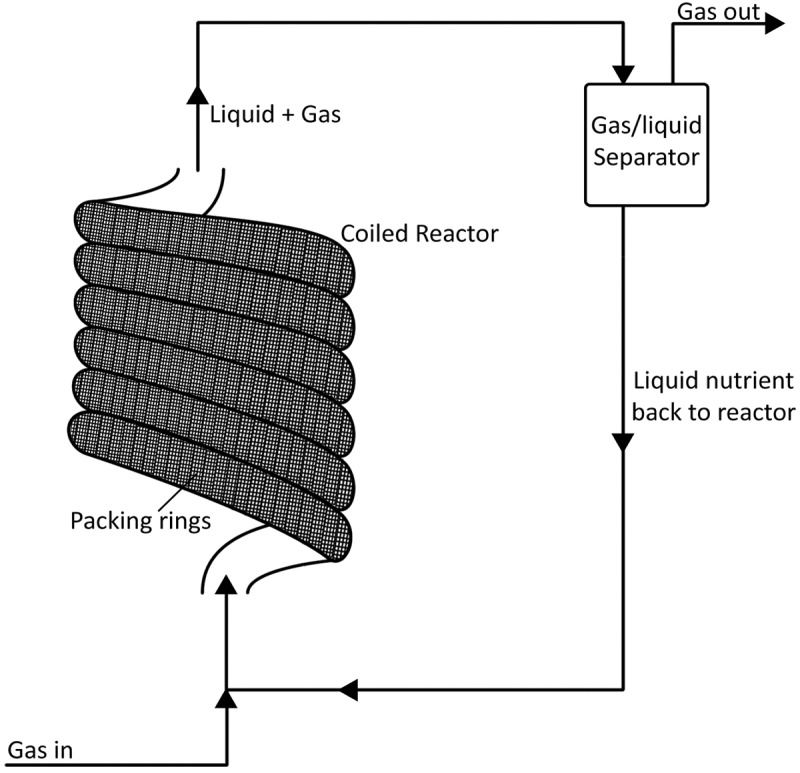
A novel BHM system using tubular reactor setup of narrow diameter pipe and packed fixed biofilm.

A novel system is presented by Savvas et al., [66] depicted in Figure 7 (adapted from Savvas et al. [66]). A hose-like reactor was set up, packed internally, creating a high surface area where archaea lined the path of the gases throughout the length of the hose reactor. The reactor was first aligned vertically (Figure 7 rotated 90°) with alternating columns of liquid and gas pockets, due to the rise and fall of coils. The complex travel path allowed for alternating stages of gas-liquid transfer. This allows for a plentiful supply of nutrients to the archaea during the rise (liquid) phase, while a large interface of gas-to-archaea during the descending loop phase. The reactor consists of a 7 meter pipe, in a six-loop configuration, with packing wheels throughout. The reactor was later orientated horizontally, drained of liquid and operated with gas recirculation (Figure 7), with pulse (10ml/min) liquid addition for moisture and nutrient provision for the biofilm. The small diameter (13 mm) of the reactor allowed the liquid to adhere to the inner surface of the reactor through surface tension forces, allowing for a uniform nutrient dispersal to archaea. The reactor was operated at four different gas loading rates, with a maximum gas throughput of 230 L/L_VR_/day with 50% CH_4_ and a MER of 40 L/L_VR_/day, the highest rate achieved by a fixed film biomethanation system of this scale. As the experimental setup is part of a fixed film system, the data for the experiment are included in Table 7. The performance of this system is noteworthy, with MER ranges of 20–39 L CH_4_/L_vr_/day at 98-50% purity, respectively. A larger-scale operation of this test is required as the experimental reactor volume measured only 0.75L.

#### 2.6.5. Soil-based fixed film reactors

Work conducted by Alitalo et al. [79] utilized two solid-state bioreactors configured in series for the BHM of H_2_ and CO_2_ in a fixed-bed reactor. The reactor consisted of a polypropylene pipe with a 75 mm diameter and 500 mm height, with an effective volume of 4 L across both reactors. The bottom 10 cm of the reactor was comprised of pebble stones while the remainder was filled with solid support comprised of vermiculite, perlite, wood ash, hydrated cobalt sulfate and hydrated nickel chloride. The specific surface area was 37.44m^2^/g. A maximum MER rate of 6.35 L CH_4_/L_vr_/day at a residence time of 144 h was achieved. In comparison, Burkhardt et al. [41] achieved 1.2 L CH_4_/L_vr_/day at a residence of 4 h with a reactor area of 305m^2^/m^3^, reaching 20% of MER at 3% of the retention time achieved by Alitalo et al. [79]. While these reactors did not produce large volumes of CH_4_, the use of aerated soil is an interesting and novel alternative to custom, plastic packing. However, an issue with this type of packing may arise due to settling of soil, formation of paths of least resistance and dead volume. As a result, modern packing may be a more suited option to promote gas dissolution and archaeal distribution.

Jee et al. [81] documented a reactor packed with granular diatomaceous earth clay as a granular support material of varied size. Tests were carried out with numerous particle sizes, where 2–3 mm particle size proved most productive as a packing material. The diatomaceous earth clay led to a consistent MER of approximately 127 L/L_vr_/day at 15.5% CH_4_ at the outlet. The same clay at 5–6 mm diameter facilitated a MER of under 90 L/L_vr_/day. Using the better performing packing, the reactor height and volume were increased and operation resumed; methane production peaked at 58% CH_4_ purity and a MER of 122.7L/L_vr_/day. The purity of the gas however steadily decreased over the course of 160 h to a lower value of 34% CH_4_ at the outlet. Jee et al. [81] hypothesized the steady decline in gas purity was due to channeling of substrate flows by the surplus cell biomass, decreasing the interference area between gas and archaea. The paper reports such high MER rates due to the inclusion of microbial strains, enriched culture medium and increased surface area, maximizing gas to archaea interference. While these results are impressive, the volumes of the reactors were small, at approximately 0.083 L and 0.136 L. As such, scaling up may yield different results. Due to soil properties, paths of least resistance may form, thus essentially rendering the majority of the soil bed a dead volume. An interesting alteration to this reactor is the injection of gas in the top of the reactor, forcing the gases to be pushed down through the fixed film and exit through the bottom. Traditional systems typically function in the opposite direction. Such a method is possible due to the reactor being primarily soil packed, allowing for gases to pass through the soil packing, as the liquid is not predominant within the reactor space as in traditional systems.

#### 2.6.6. Hollow fiber reactors (HFR)

**Figure 8.**
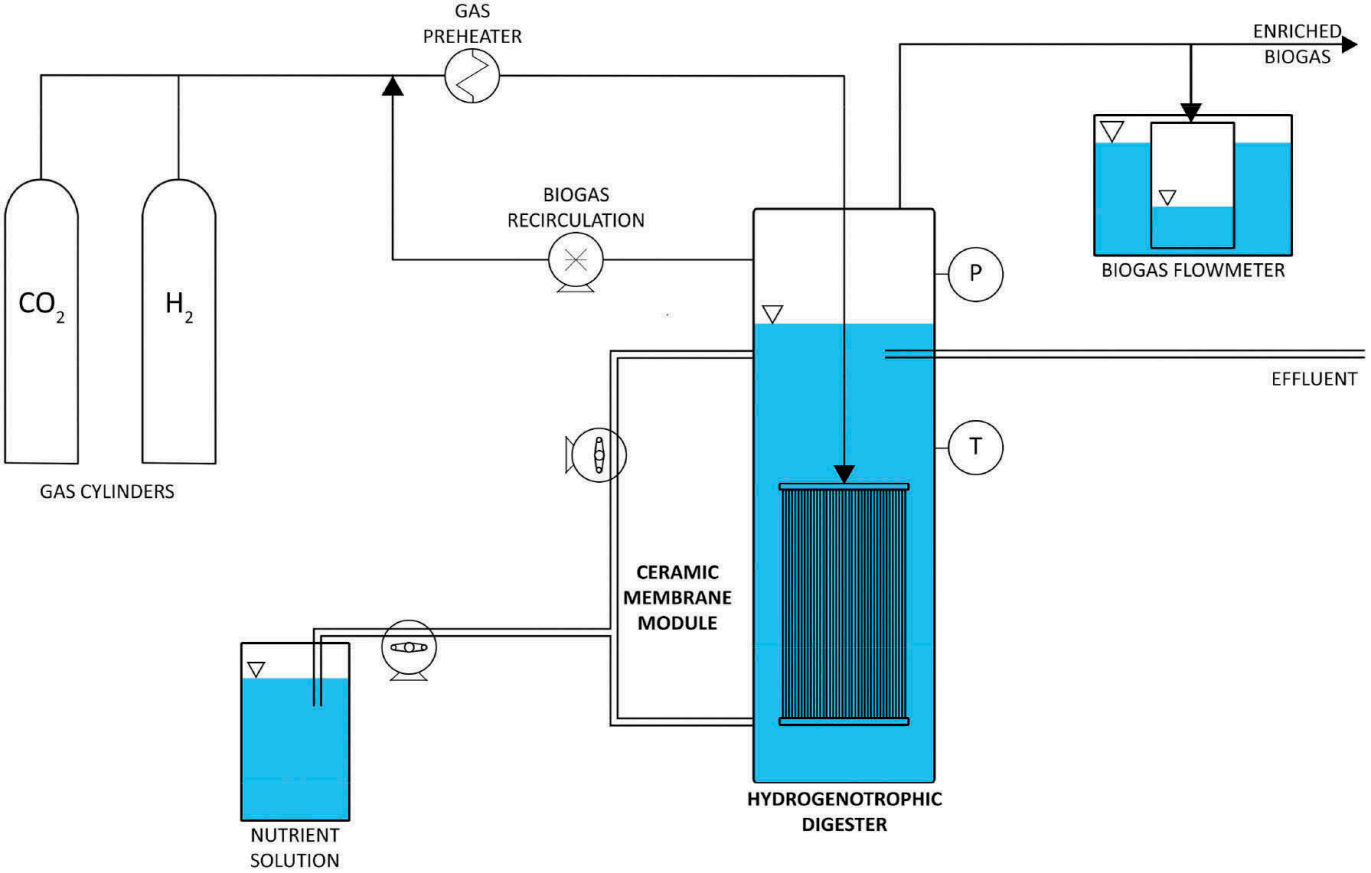
Diagram of a membrane bio-reactor experimental set-up.

Membrane bioreactors are based on specially designed ceramic membranes, typically referred to as hollow fiber membranes. An example of such a system is shown in Figure 8 (adapted from Díaz et al. [61]). These provide a barrier between the reactor liquor and gas supply. The fiber membrane comprises of many fibers, from which gas is forced through small pores, diffusing straight into the surrounding liquid. This process relies on the porosity of the fiber membranes. Designs range from hydrophilic to hydrophobic, which represent a wide variety of hollow fiber materials. Hydrophilic fibers require the membranes to undergo a process called wetting, with membranes absorbing water into the pores. This process ensures no gas bubbles are present in the membrane before commissioning [82]. While hollow fiber membranes provide instantaneous gas to liquid mass transfer, the flow rates of the system are limited due to the porosity and relatively small surface area of these membranes. There are a number of merits that this system offers; high purity of exit gas, compactness and easier automation [83]. Disadvantages vary from high capital and operation costs from membrane fouling, relating to the build-up of biofilm on the membrane over the system lifespan, decreasing the operational efficiency of the system. A study by Díaz et al. [61] demonstrated the utilization of this system, with significant results achieved. The system produced a MER of 7 L CH_4_/L_vr_/day at 95% H_2_ utilization with a 1 h retention time. A noteworthy result was the lack of a biomass film found on the membrane of the module as compared to previous studies. This occurrence is attributed to the high recirculation rates of gas. Other literature setups involving HFR presented yields of 40–50 L CH_4_/L_vr_/day at 25-14% CH_4_ purity, respectively. A comparison of HFR used in BHM is shown in Table 8. Once again, the trade-off between purity and MER is evident as higher throughputs allow for higher MER rates, but lower the purity of the exiting gas.

**Table 8.** Comparison of Hollow membrane reactors in BHM throughout literature.

	*Reactor Configuration*		*H_2_*	*CO_2_*		*CH_4 out_*	*Pressure*	*Temp.*	*Reactor Vol.*	*MER*	*Retention Time*	k_L_a		*Rate of mixing*		Gas recirculation	*Enriched Culture*			
*Type*	*(in/ex-situ)*	*pH*	*(L/L_vr_/min)*	*(L/L_vr_/min)*	*H_2_/CO_2_ Ratio*	*(%)*	*(Barg)*	*(°C)*	*(L)*	*(L CH_4_/L_vr_/day)*	h	/h	Method of k_L_a Increase	*(L/min)*	*(rpm)*	(L/min)	*(y/n)*	*Archaeal strain*	*Unit normalized to*	*Author*
HFM R	Ex-situ	7.2	0.0070	0.0018	4 : 1	84	0	55	31	2.0	2.80	30	HFM + Recirculation	-	-	0.07	No	-	STP	[61]
HFM R	Ex-situ	7.2	0.0140	0.0035	4 : 1	75	0	55	31	3.8	1.37	155	HFM + Recirculation	-	-	1.12	No	-	STP	[61]
HFM R	Ex-situ	7.2	0.0210	0.0052	4 : 1	68	0	55	31	5.7	0.91	180	HFM + Recirculation	-	-	1.12	No	-	STP	[61]
HFM R	Ex-situ	7.2	0.0210	0.0052	4 : 1	79	0	55	31	6.9	1.01	240	HFM + Recirculation	-	-	1.67	No	-	STP	[61]
HFM R	Ex-situ	7.2	0.0314	0.0078	4 : 1	65	0	55	31	9.5	0.64	420	HFM + Recirculation	-	-	3.35	No	-	STP	[61]
HFM R	Ex-situ	7.2	0.0174	0.0044	4 : 1	82	0	55	31	5.8	1.21	205	HFM + Recirculation	-	-	1.51	No	-	STP	[61]
HFM R	Ex-situ	7.2	0.0279	0.0070	4 : 1	78	0	55	31	8.8	0.74	430	HFM + Recirculation	-	-	3.35	No	-	STP	[61]
HFM R	Ex-situ	7	0.0187	0.0047	4 : 1	25	0	65	0.104	40.3	-		HFM	-	-	0	Yes	M. Thermoautotrophicus	N/A	[84]
HFM R	Ex-situ	7	0.0320	0.0080	4 : 1	14	0	65	0.104	50.7	-		HFM	-	-	0	Yes	M. Thermoautotrophicus	N/A	[84]
MBR	Ex-situ	7.4	0.0069	0.0017	4 : 1	79	0	55	60	2.4	3.10	77	Ceramic tubular membrane	-	-	8	No	-	STP	[58]
MBR	Ex-situ	7.4	0.0139	0.0035	4 : 1	55	0	55	60	4.3	1.46	87	Ceramic tubular membrane	-	-	8	No	-	STP	[58]
MBR	Ex-situ	7.4	0.0139	0.0035	4 : 1	79	0	55	60	4.8	1.55	166	Ceramic tubular membrane	-	-	12.3	No	-	STP	[58]
MBR	Ex-situ	7.4	0.0208	0.0052	4 : 1	79	0	55	60	7.1	1.03	268	Ceramic tubular membrane	-	-	12.3	No	-	STP	[58]
HFM R	In-situ	7.61	0.0006	0.0003	4 : 1	78	0.32	55	1	0.9	585.37		Magnetic stirrer	-	150	0	No	-	STP	[73]
HFM R	In-situ	7.9	0.0010	0.0003	4 : 1	90	0.56	55	1	0.9	45.89		Magnetic stirrer	-	150	0	No	-	STP	[73]
HFM R	In-situ	8.31	0.0012	0.0003	4 : 1	96	0.75	55	1	0.9	28.74		Magnetic stirrer	-	150	0	No	-	STP	[73]

- Retention time values presented in papers are noted (**Bold**, underlined and *italic*), but where missing, retention time according to Voelklein et al. [11] calculated to allow comparison.

### 2.7. System key parameter comparison

As discussed, systems throughout literature vary substantially in their capability to achieve a high MER and high purity output gas. The following subsections compare these systems to show the development of each of the technologies, as well as the current state of the art.

#### 2.7.1. Comparison of methane evolution rate across reactor systems

**Figure 9.**
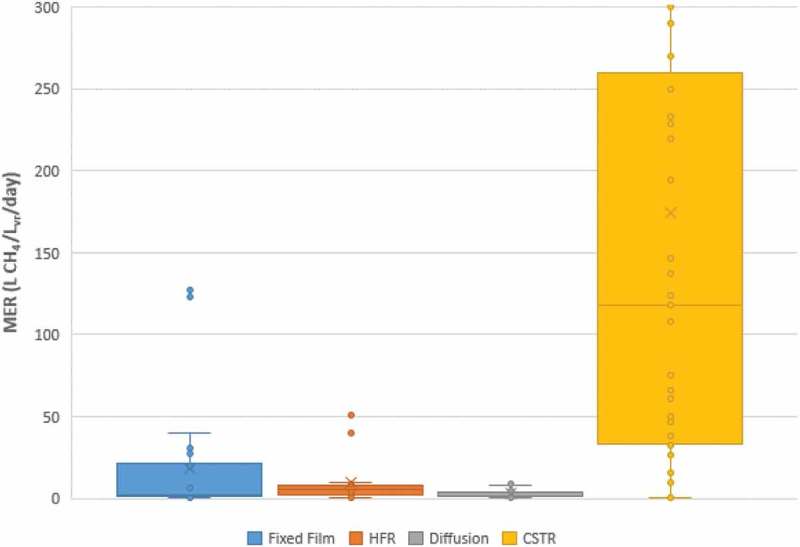
Comparison of MER across reactor systems.

Across the systems investigated, MER is a parameter that is shown to vary significantly. With reference to Figure 9, the use of CSTRs generates significantly higher MER than the other reactor systems; however, there is a substantial variation in the MER values achieved in literature. Additionally, it is important to take into account the early development stage of the technologies. From the observation of data in Tables 5–8, in the presence of microbial strains such as Methanothermobacter thermoautotrophicus the resulting methane production rates are substantially higher. It is evident that the CSTR technology is the predominant system for assessment of pure strain cultures. This highlights that even minor improvements to the different BHM systems can cause significant jumps in the efficiency as illustrated by the MER. The use of pure cultures is usually omitted due to the additional complications this causes in laboratory-scale experiments.

#### 2.7.2. Comparison of retention time across reactor systems

**Figure 10.**
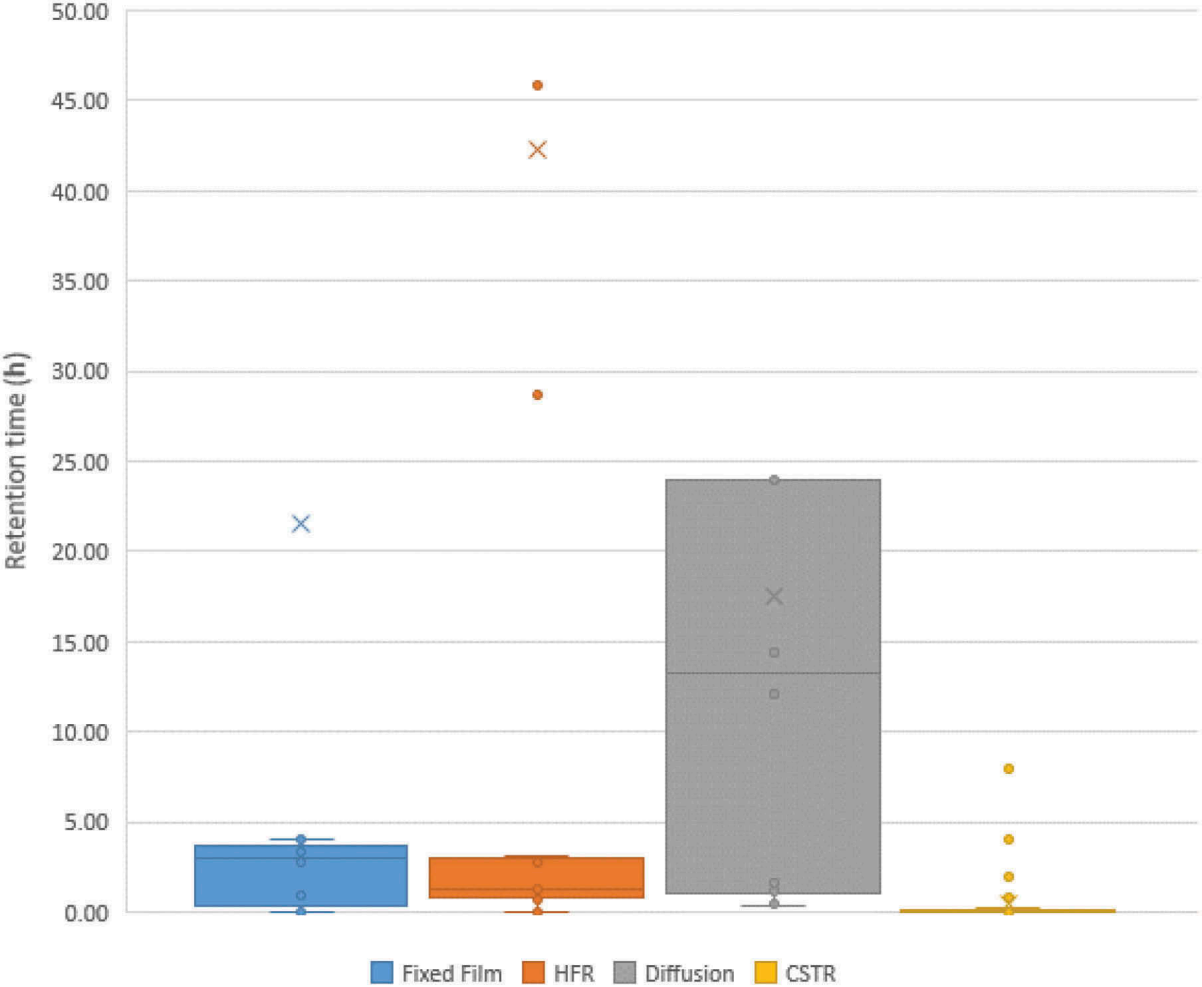
Comparison of retention times across reactor systems.

Figure 10 highlights the variations in retention time across reactor systems. Low retention times in BHM systems are indicative of a higher reactor efficiency, suggesting shorter travel paths and, as such, more compact and potentially cheaper systems resulting in more cost-effective renewable gas production. Recirculation is a simple method to prolong these travel paths within a compact system but can also be an indication of the lower performance of a reactor system and earlier development stage. Diffusion-based systems are found to have the highest retention times of the different BHM systems assessed. There was however a significant variation in the retention times. CSTR-based BHM systems typically had the lowest retention times. This highlights the performance of CSTR reactors, and also a more mature state of this reactor system when compared to others assessed.

### 2.8. Analysis of relationship between key operational parameters

As indicated in the analysis and comparisons, cross-system appraisal is difficult at an experimental level. This is mainly due to the various technology development stages. Most research has focused on the use of CSTRs for BHM, due to the fact that this is the most mature reactor technology, and also the simplest. As a result, a more system-by-system comparison should be carried out across reactor set-ups, irrespective of the system implemented. The following sections seek to compare several parameters collated from literature.

#### 2.8.1. Relationship between retention time and methane evolution rate

**Figure 11.:**
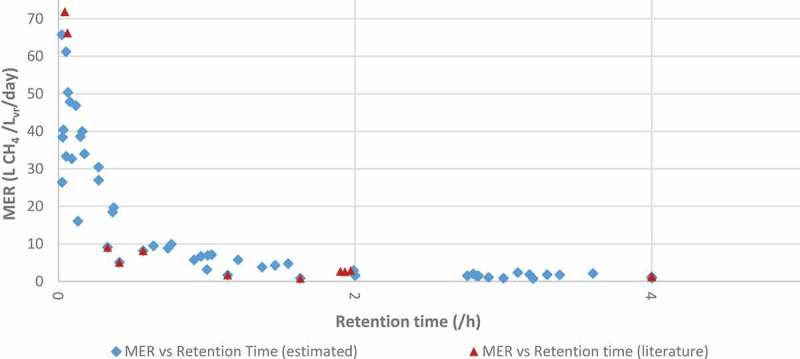
Relationship between MER and retention times.

From the analysis of the data compilation in Tables 5–8, a relationship was sought between methane evolution rate and retention time in the BHM reactors. Upon plotting the data, the relationship, as documented in Figure 11 was found. Instead of the expected increase in MER with longer retention time, the opposite appeared to occur. The authors believe that this is an indication of the system inefficiency at high retention time, in which gas requires longer residence times to be converted in the reactor system. This relationship suggests that smaller more efficient reactor systems will result in the generation of more cost-effective green gas.

#### 2.8.2. Relationship between CH_4_ concentration and methane evolution rate

**Figure 12.:**
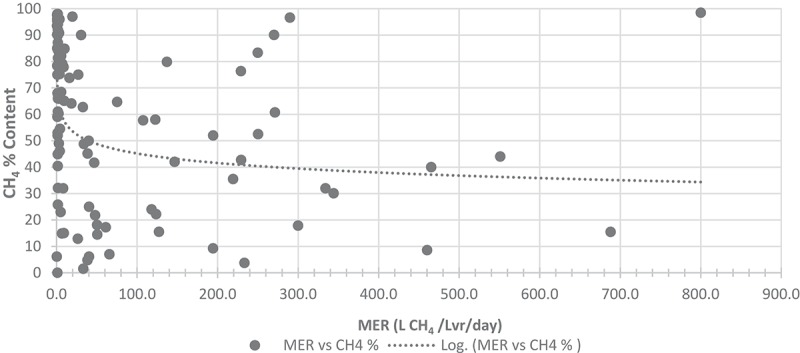
Relationship between MER and CH_4_% content.

Analysis of the data in Tables 5–8 reveals a weak relationship (Figure 12) between CH_4_ concentration and MER, a concept supported by Lecker et al. [14]. While the relationship is weak, the earlier discussion of higher MER levels resulting in lower CH_4_ percentages in the outlet gas supports the existence of this relationship. A conclusion can be made that lower MERs tend to yield higher purity exiting gas. Few studies have been able to maximize the values of these two variables. At a full demonstration scale, ‘Electrochaea GmbH’ [26] was capable of elevating the levels of both of these values, likely due to the refining of the full-scale methanation system, with the use of pure cultures, high agitation rates, high gas throughputs, low retention times and high pressures. Optimal systems with ideal configurations require complex maintenance as imbalances and oversight may lead to reactor contamination and sub-optimal production rates.

#### 2.8.3. Relationship between methane evolution rate and volumetric gas-liquid mass transfer coefficient k_L_a

**Figure 13.**
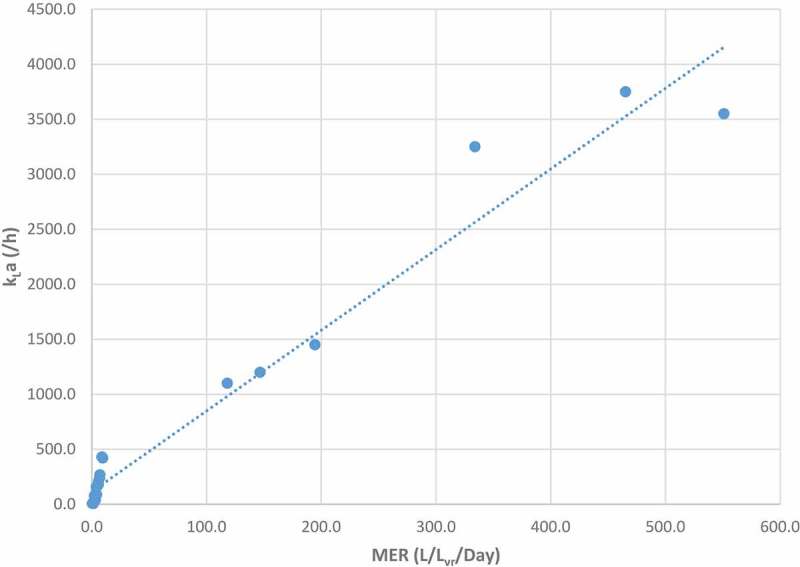
Comparison of MER vs k_L_a data in literature.

Using available data in the literature on k_L_a and MER, an exercise was carried out in an effort to observe any relationship between the two parameters. The literature data compiled in Table 9 allowed for the production of Figure 13. A cursory examination shows that there is a relationship between MER and k_L_a, as expected. Increased MER coincided with increased k_L_a values. However, as outlined previously, data on exact k_L_a measurement are limited, more data and laboratory research are required to elucidate the exact significance and nature of this relationship.

**Table 9.** MER vs k_L_a comparison.

*MER*	k_L_a	
*(L CH_4_/L_vr_/day)*	/h	*Author*
465.3	3750	[48]
551.0	3550	[48]
334.0	3250	[48]
194.5	1450	[48]
146.7	1200	[48]
118.1	1100	[48]
8.8	430	[61]
9.5	420	[61]
7.1	268	[58]
6.9	240	[61]
5.8	205	[61]
5.7	180	[61]
4.8	166	[58]
3.8	155	[61]
4.3	87	[58]
2.4	77	[58]
3.2	39.9	[69]
2.0	30	[61]
1.6	20.5	[69]
1.6	19.9	[69]
1.2	16.1	[73]
1.2	11.8	[73]
0.9	11.4	[69]
0.5	6.8	[69]
1.429	6.62	[73]

### 2.9. Full-scale demonstration plant

**Figure 14.**
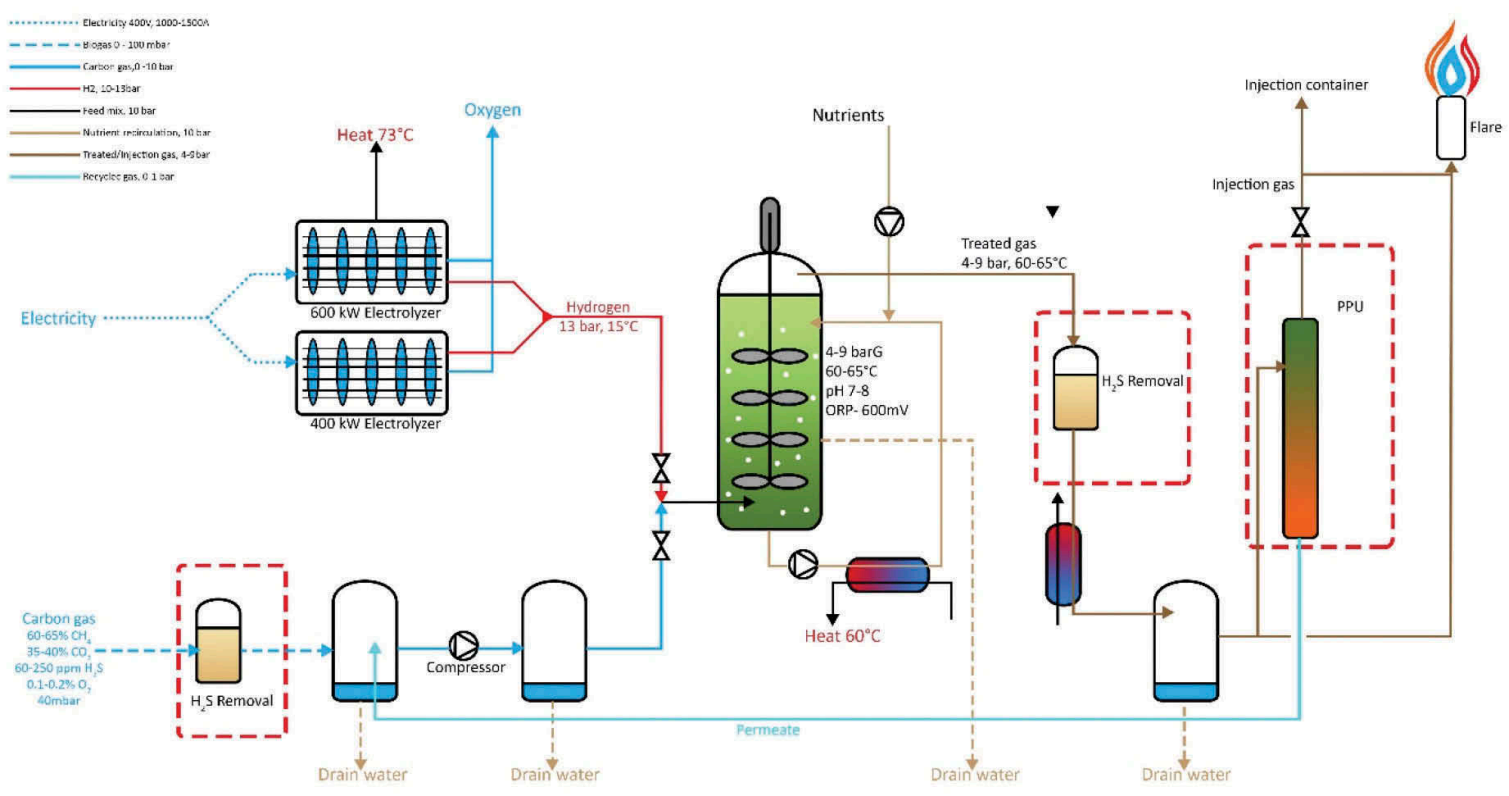
Electrochaea plant schematic adapted from the plant technical report[26].

To the authors' knowledge, ‘Electrochaea GmbH’ is the first full-scale methanation plant. It operates at a full load capacity of 1.502 MW (raw biogas case) in Copenhagen, Denmark. Personal communication with the company and conference presentations indicate MER values of 800 L CH_4_/L_vr_/day, at 99% CH_4_ content and estimated gas residence times of 3 min [85]. The plant is heated only up to 50°C and the exothermic reaction of archaea brings the reactor temperature up to 60°C. The state-of-the-art plant yields 0.126 MWh_th_ thermal energy (counter-flow heat exchanger) generated from the bioreactor process, 0.544 MWh_th_ in the form of upgraded CO_2_ and H_2_, and 0.832 MWh_th_ in the form of injected methane contained in the raw biogas. The additional yield equates to a methane yield increase of over 65%. The report proposed scenarios for sourcing CO_2_; purchasing raw biogas (CO_2_/CH_4_ mixture) from a nearby plant during operational hours, or, using CO_2_ obtained from scrubbing raw biogas and injecting into the upgrading plant along with H_2_ for upgrading. Hydrogen is sourced from an electrolyzer on-site which operates during periods of excess electrical energy, capitalizing on the availability of ‘waste’ electricity, based on Denmark’s high renewable energy access in the form of wind. This allows for the electrical energy to be stored in gaseous form in the gas grid. Oxygen is also produced in the electrolyzer; however, due to the intermittent generation and relatively low financial value of O_2_, it is determined that the capture and use of the O_2_ are not profitable. However, consideration was made to use the O_2_ in aeration of the nearby wastewater treatment facility. The BHM reactor is designed as a 9 m tall CSTR with a 0.72 m ID (estimated), with four impellers, mixing at a rapid speed, and gas injection of H_2_ and CO_2_ at the bottom of the tank. A plant layout schematic is depicted in Figure 14. The shear forces exerted by the impellers cause a breakup of bubbles, and subsequent impellers along the height of the reactor ensure the maintenance of small bubble size. The reactor operates at 60-65°C and 4–9 bar pressure. Due to the height of the reactor, pressure on the bottom will be 1 barg higher than the top. Industry examples suggest the agitation requires 1.6 W/L_reactor_, and with an estimated total reactor volume of 3750 L this equates to 5.70kW for CSTR agitation. This matches closely to the industry technical reports [26,85,86]. In terms of energy demand, Electrochaea (approximately 0.32 kWh/Nm^3^ raw biogas) is similar to traditional gas purification systems whose energy demand can range significantly (Water scrubber: 0.25–0.3 kWh/Nm^3^; Chemical scrubber: 0.05–0.15 kWh/Nm^3^; Membrane separation: 0.18–0.2 kWh/Nm^3^; Pressure Swing Absorption 0.23–0.30 kWh/Nm^3^; Cryogenic upgrading; 0.76 kWh/Nm^3^; Organic physical scrubber: 0.2–0.3 kWh/Nm^3^).

Biologically, a patented, isolated strain of Methanothermobacter Thermoautotrophicus UC 120,910 is used in a pure culture. When gases are injected, they undergo screening to ensure no contaminants are brought into the main reactor. Nutrient media are periodically injected. The environment has a pH range of 7–8 and an Oxidation-Reduction Potential (ORP) of 600mV, corresponding to a 0 coliform count per 100 ml of water. A Life Cycle Analysis (LCA) baseline performance shows a 75% CO_2_ emissions reduction compared to fossil fuel–derived natural gas [26]. The reactor is capable of producing grid quality gas, at pressures greater than 6 barg. The only additional step for grid injection is the removal of water vapor through drying.

## 3. Conclusions

The energy transition will raise dilemmas to challenge future energy systems. One major challenge is energy storage and the matching of energy production with energy demand. Another being, difficult to decarbonize sectors such as haulage. The circular economy combination of hydrogen produced via electrolysis of curtailed/constrained electricity, biogas production from the digestion of organic waste upgraded to gas grid specification through the reaction of biogas and the aforementioned hydrogen provides a decentralized form of energy storage, and an energy vector for haulage.

The BHM systems reviewed have challenges. In-situ systems need to prevent complicated inhibition pathways due to hydrogen partial pressures. To produce a financially sustainable green gas ex-situ methanation system, optimization of methane evolution rate through maximization of gas transfer rates, while optimizing methane content in the produced gas is required. Retention time was viewed as a method of elevating MER rates by extending the travel path of a unit of gas through the upgrading system. While this is a valid option for research, long retention times of gases imply an inefficient system producing expensive green gas. As such, prolonged retention times indicate the low efficiency of BHM systems.

The ex-situ technology is at a low technology readiness level. Some literature demonstrates high MER values and high volumetric gas throughput utilizing high rate mixing and agitation (1500rpm), yet the percentage CH_4_ in the outlet gas remains low (15.5%) [15]. The energy demand of high rate mixing can be substantial [15,86]. CSTRs appear to be the reactor system most capable of achieving elevated MER. However, this is not indicative of the reactor configuration but rather system optimization (such as patented strains of pure culture) that has been carried out with CSTR reactors. As such, novel systems can currently be portrayed as less successful but may have strong benefits in future energy systems if fully optimized. Indeed, it is suggested by the authors that lower MER in recent publications indicate that optimization has been delayed, as novel reactor systems are studied.

There are evident gaps in the state of the art with regards to system efficiency and optimization. This review has identified that a significant gap may be in the use of diffusion based technology for H_2_ dissolution. More work is required to ascertain where diffusion fits in the bigger picture of BHM. In addition, the lack of research into solubilization of H_2_ leaves extensive gaps in the state of the art in BHM relating to H_2_ diffusion through micro and nano-pore diffusers. Research of the smallest possible bubble diameters achievable in reactor liquors is open to exploration. This would require investigation on liquor viscosity, shear forces, particulate presence, chemical effects; and finally if viable, application of the learned knowledge on an experimental ex-situ methanation reactor would provide a novel research opportunity. Finally, development of a method of evaluation for k_L_a would allow for a more time efficient method of evaluating k_L_a, a key reactor performance parameter when compared to laboratory procedures. This may allow for a better comparison between reactor configurations when coupled with MER and retention time.

## Highlights

- Novel technologies are essential for optimal biomethanation.
- Retention time is a poor indication of biomethanation system efficiency.
- Bubble diffusion in biomethanation presents a gap in the state of the art.
- Reactor gas-liquid transfer data is scarce, showing gaps in the state of the art.
