# Supplementary material for: Biological hydrogen methanation systems – an overview of design and efficiency
Source: Bioengineered. 2019 Nov 3;10(1):604–34. doi: 10.1080/21655979.2019.1684607 (PMC6844437; doi:10.1080/21655979.2019.1684607)
Supplement: Supplemental Material [file kbie-10-01-1684607-s001.docx]

Biological Hydrogen Methanation Systems – An overview of design and efficiency.

**Authors**

Davis Rusmanis^[[1]](#footnote-1),^^[[2]](#footnote-2)^, Richard O’Shea^a,b,^^[[3]](#footnote-3)^, David M. Wall^a,b^, Jerry D. Murphy^a,b^

# Supplementary data

The purpose of Box S1 is to provide a comparison for use of methane (CH_4_) and hydrogen (H_2_) as an energy carrier gas. An energy transmission of 1GW_th_ was chosen as an arbitrary value of energy required to be transmitted

**BOX S1**

|  |  | Methane | Hydrogen |
| --- | --- | --- | --- |
| Density | kg/m^3^ | 6.585 | 0.8085 |
| Viscosity | CP | 1100 | 880 |
| LHV | MJ/kg | 50 | 119.9 |

Gas grid pressure: 1MPa

Temperature: 25°C

Energy transmission: 1,000MW_th_ (Arbitrary number chosen for comparison.)

**Hydrogen**

Mass Flow:

$$\frac{1000}{119.9} =8.34\frac{kg}{s}$$

Volume Flow:

$$\frac{8.34}{0.8085}=10.315\frac{m^{3}}{s}$$

Pressure Drop:

$$=4.0156\frac{kPa}{{km}_{pipe}}$$

Specific work:

$$\frac{4.0156}{0.8085}= 4.967\frac{kJ}{{kg}_{H2} . {km}_{pipe}}$$

Power:

$$4.967*8.34=41.42\frac{kW}{{km}_{pipe}}$$

**Methane**

Mass Flow:

$$\frac{1000}{50}=20\frac{kg}{s}$$

Volume Flow:

$$\frac{20}{6.585}=3.037\frac{m^{3}}{s}$$

Pressure Drop:

$$1.477\frac{kPa}{{km}_{pipe}}$$

Specific work:

$$\frac{1.477}{6.858}=0.224 \frac{kJ}{{kg}_{CH4} . {km}_{pipe}}$$

Power:

$$0.224*20=4.48\frac{kW}{{km}_{pipe}}$$

1. MaREI Centre, Environmental Research Institute (ERI), University College Cork (UCC), Ireland. [↑](#footnote-ref-1)
2. School of Engineering, UCC, Ireland. [↑](#footnote-ref-2)
3. Corresponding author at: School of Engineering, University College Cork, Cork, Ireland. E-mail address: [richard.oshea@ucc.ie](mailto:richard.oshea@ucc.ie) (Richard O’Shea) [↑](#footnote-ref-3)
